# Low dose radiation and cancer in A-bomb survivors: latency and non-linear dose-response in the 1950–90 mortality cohort

**DOI:** 10.1186/1476-069X-6-1

**Published:** 2007-01-18

**Authors:** Greg Dropkin

**Affiliations:** 1 Flat 5, 32 Sheil Rd., Liverpool L6 3AE, UK

## Abstract

**Background:**

Analyses of Japanese A-bomb survivors' cancer mortality risks are used to establish recommended annual dose limits, currently set at 1 mSv (public) and 20 mSv (occupational). Do radiation doses below 20 mSv have significant impact on cancer mortality in Japanese A-bomb survivors, and is the dose-response linear?

**Methods:**

I analyse stomach, liver, lung, colon, uterus, and all-solid cancer mortality in the 0 – 20 mSv colon dose subcohort of the 1950–90 (grouped) mortality cohort, by Poisson regression using a time-lagged colon dose to detect latency, while controlling for gender, attained age, and age-at-exposure. I compare linear and non-linear models, including one adapted from the cellular bystander effect for α particles.

**Results:**

With a lagged linear model, Excess Relative Risk (ERR) for the liver and all-solid cancers is significantly positive and several orders of magnitude above extrapolations from the Life Span Study Report 12 analysis of the full cohort. Non-linear models are strongly superior to the linear model for the stomach (latency 11.89 years), liver (36.90), lung (13.60) and all-solid (43.86) in fitting the 0 – 20 mSv data and show significant positive ERR at 0.25 mSv and 10 mSv lagged dose. The slope of the dose-response near zero is several orders of magnitude above the slope at high doses.

**Conclusion:**

The standard linear model applied to the full 1950–90 cohort greatly underestimates the risks at low doses, which are significant when the 0 – 20 mSv subcohort is modelled with latency. Non-linear models give a much better fit and are compatible with a bystander effect.

## Background

Studies of the Japanese A-bomb survivors cohort have been the key source of radiation risk estimates used to establish environmental and occupational protection standards [1,2] despite the differences between instantaneous exposure in war to gamma and neutron irradiation, and chronic low dose occupational or public exposure in general or to alpha particles. There is an unresolved debate [3] on whether Excess Relative Risk (ERR) increases linearly with dose across a very large range of exposures including the low dose range.

Currently the International Commission on Radiological Protection (ICRP) recommends an annual occupational dose limit of 20 mSv (whole body dose), and many researchers believe that doses below this level have little or no impact on human health. The ICRP risk estimates are strongly based on analyses of the Japanese data for the full dose range assuming a linear dose response. On the other hand in the Life Span Study Report 12 (henceforth LSS12) Pierce et al. [4] adopted a linear ERR model for the Japanese data but found some evidence of a non-linear convex dose-response at lower doses for the category of all-solid cancers combined.

Around one third of the data in the 1950–90 mortality cohort analysed in LSS12 concerns persons who received 0 – 20 mSv instantaneous exposure in 1945. There is no reason to assume any particular relation between dose response in this region and across the entire follow-up. Instead, the low dose data may be analysed in its own right with or without assuming a linear dose response.

In experiments at cell level, low dose radiation induces significant and non-linear effects on micronuclei, chromosome rearrangements and instability, point mutations, and changes in cell cycle protein levels. Studies reviewed by Mothersill and Seymour [5] show low doses have large effects on cells whose nuclei, or even the affected cells themselves, need not be directly traversed by radiation (bystander effects). Some studies find responses rising rapidly with dose and then reaching a plateau. Whilst these early non-linear cellular events may be critical for tumour formation, the human body's defences may also greatly alter the initial dose response or dilute its significance, if the analysed outcome is a cancerous tumour emerging decades later and eventually identified as cause of death.

I analyse the five leading cancer sites (stomach, liver, lung, colon, and uterus) and the grouped category of all-solid cancers in the 0 – 20 mSv portion of the 1950 – 90 Japanese mortality cohort. Follow-up began 5 years after exposure but rather than assume this lag to be optimal, latency is estimated for individual cancers by fitting models which include a variable latent period. I test linearity in the dose response by modelling with two nested non-linear models, each of which contains the linear model.

## Methods

Life Span Study mortality cohort data (1950–1990) were obtained from the Radiation Effects Research Foundation [6] via the Comprehensive Epidemiologic Data Resource [7]. The file r12canc.dat includes anonymous information on a cohort of 86 572 survivors, presented as 16 612 grouped data cells cross-tabulated by city, sex, total shielded kerma, exposure age and attained age categories, and dose category. Cell data includes a weighted adjusted colon dose, representing an average of individual instantaneous ("flash") doses weighted by person-years within the cell (and assuming neutron Relative Biological Effectiveness RBE = 10). Mean age-at-exposure, mean attained age, and deaths from specific cancers or grouped cancer categories are also shown for each cell. Liver cancer (ICD9 155(0,1,2)) includes both deaths attributed to primary liver cancer, and deaths which were not specified as primary or secondary liver cancer. Cancer of the uterus refers to ICD9 180–182, including cervical cancer (ICD9 180).

The data uses the DS86 dosimetry [8], but a limited comparison with the current DS02 dosimetry [9-11] is undertaken.

Subcohorts are defined by restricting weighted adjusted colon dose as given in the cell data. The 0 – 20 mSv subcohort has 3011 cells comprising 1690391.75 person-years (p-y) observation. This subcohort consists of exactly those cells whose individual members received no more than 20 mSv adjusted colon dose (3009 cells have dose category 1 or 2, while two other cells each contain a single person at risk whose adjusted colon dose was 20 mSv). Other subcohorts defined below lack such simple interpretation.

Specified cancer deaths in the i^th ^cell are assumed to be Poisson distributed with expected value λ_i_T_i _where T_i _is p-y observation and λ_i _depends on control variables and a lagged dose D_φ _defined for each cell as weighted adjusted colon dose (in 10 mSv units) if Time-Since-Exposure ≥ φ and 0 otherwise. Time-Since-Exposure = mean attained age – mean age at exposure.

The models below assume

λ = λ_0 _(1 + ERR)

λ_0 _= e(α+Σjβjyj)
 MathType@MTEF@5@5@+=feaafiart1ev1aaatCvAUfKttLearuWrP9MDH5MBPbIqV92AaeXatLxBI9gBaebbnrfifHhDYfgasaacH8akY=wiFfYdH8Gipec8Eeeu0xXdbba9frFj0=OqFfea0dXdd9vqai=hGuQ8kuc9pgc9s8qqaq=dirpe0xb9q8qiLsFr0=vr0=vr0dc8meaabaqaciaacaGaaeqabaqabeGadaaakeaacqqGLbqzdaahaaWcbeqaaiabcIcaOiabeg7aHjabgUcaRiabfo6atnaaBaaameaacqqGQbGAaeqaaSGaeqOSdi2aaSbaaWqaaiabbQgaQbqabaWccqWG5bqEdaWgaaadbaGaeeOAaOgabeaaliabcMcaPaaaaaa@3BB6@

where y_j _are control variables while ERR depends on D_φ _and parameters θ_k _(including φ) but not on control variables or their parameters.

Gender, log mean attained age, and 14 indicator variables defined by 0 ≤ age-at-exposure < 5, 5 ≤ age-at-exposure < 10,... and 65 ≤ age-at-exposure < 70 were used as controls. Indicator variables defined from mean age-at-exposure (cell data) are identical to those defined from the age-at-exposure stratification of the dataset. An indicator defined by 70 ≤ age-at-exposure would be redundant. Log mean attained age was an adequate alternative to the use of attained age categories.

Four models were fitted:

Linear ERR = βD_φ_

Transient ERR = σD_φ _[e(−τDφ)
 MathType@MTEF@5@5@+=feaafiart1ev1aaatCvAUfKttLearuWrP9MDH5MBPbIqV92AaeXatLxBI9gBaebbnrfifHhDYfgasaacH8akY=wiFfYdH8Gipec8Eeeu0xXdbba9frFj0=OqFfea0dXdd9vqai=hGuQ8kuc9pgc9s8qqaq=dirpe0xb9q8qiLsFr0=vr0=vr0dc8meaabaqaciaacaGaaeqabaqabeGadaaakeaacqqGLbqzdaahaaWcbeqaaiabcIcaOiabgkHiTiabes8a0jabbseaenaaBaaameaacqaHgpGzaeqaaSGaeiykaKcaaaaa@358E@] with τ ≥ 0

Two-phase ERR =βD_φ _+ σD_φ _[e(−τDφ)
 MathType@MTEF@5@5@+=feaafiart1ev1aaatCvAUfKttLearuWrP9MDH5MBPbIqV92AaeXatLxBI9gBaebbnrfifHhDYfgasaacH8akY=wiFfYdH8Gipec8Eeeu0xXdbba9frFj0=OqFfea0dXdd9vqai=hGuQ8kuc9pgc9s8qqaq=dirpe0xb9q8qiLsFr0=vr0=vr0dc8meaabaqaciaacaGaaeqabaqabeGadaaakeaacqqGLbqzdaahaaWcbeqaaiabcIcaOiabgkHiTiabes8a0jabbseaenaaBaaameaacqaHgpGzaeqaaSGaeiykaKcaaaaa@358E@] with τ ≥ 0

Category ERR = β_1_(dosecat1) + β_2_(dosecat2) + β_3_(dosecat3)

The usual **Linear **model is used here with lagged dose. The Control model (ERR = 0) is nested when β = 0.

The **Transient **model gives an asymptotic linear dose response at very low doses, decaying to zero at higher doses if τ > 0. The Linear model is nested when τ = 0, while σ = 0 nests the Control model.

The **Two-phase **model gives an asymptotic linear dose response at very low doses, and a possibly different asymptotic linear response at higher doses, with an exponential transition between the two. The Transient model is nested when β = 0, while σ = 0 (or τ = 0) nests the Linear model. The Control model is nested when β = σ = 0, or when τ = 0 and β + σ = 0. ERR ~ βD_φ _when τ D_φ _>> 1, while ERR ~ (β + σ)D_φ _when τ D_φ _<< 1. The ratio of asymptotic slopes is **R **= 1 + σ/β.

**Figure 1:**
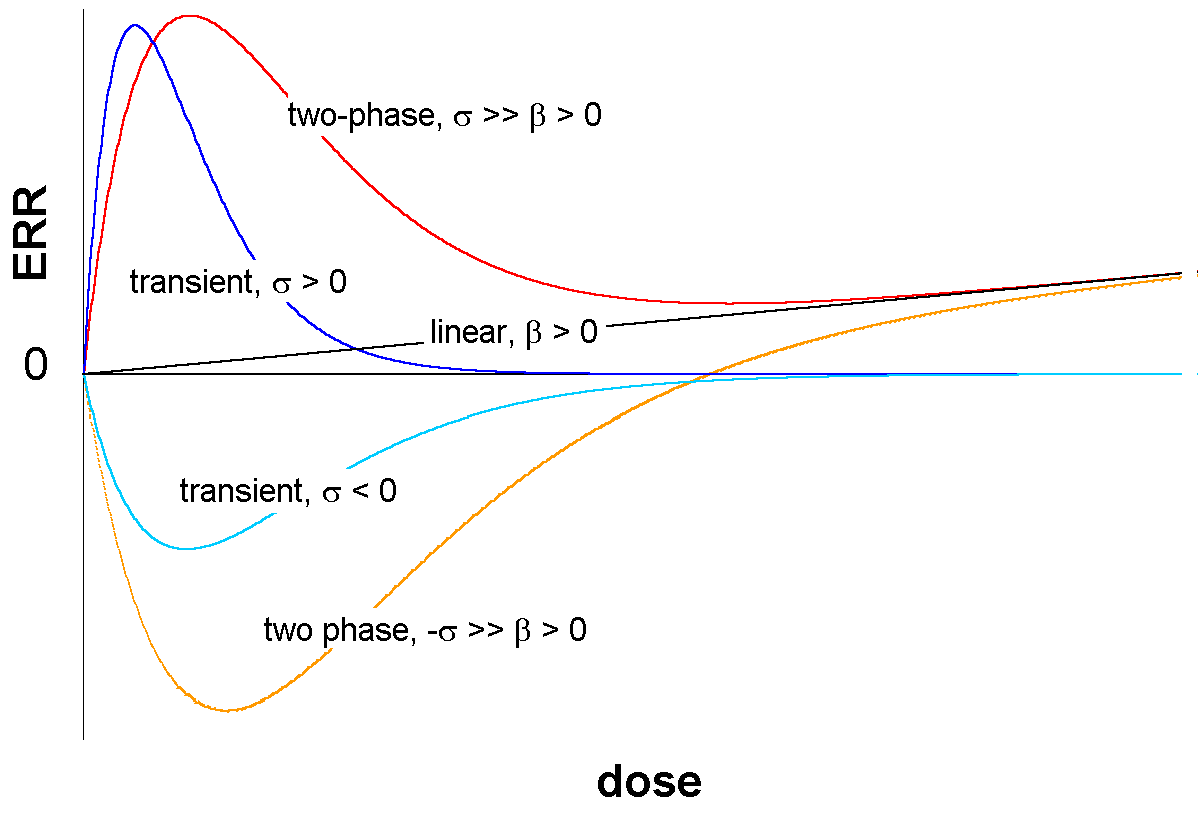
**Dose response prototypes for linear, transient, and two-phase models**. Typical dose response curves for the linear model ERR = βD where ERR is Excess Relative Risk and D is a dose variable (arbitrary units), the transient model ERR = σDe^(-τD)^, and the two-phase model ERR = βD + σDe^(-τD)^. Here β, σ, τ are parameters with τ ≥ 0. The linear model is shown with β > 0. Two versions of the transient model are shown, with σ > 0 and with σ < 0. The transient model converges to 0 as dose → ∞. Two versions of the two-phase model are shown, with σ >> β > 0 and -σ >> β > 0; the first version occurs when β > 0 and σ/β > e^2 ^~ 7.39. Other possibilities (not shown) lack local maxima and minima, but the two-phase model always converges to the linear model as dose → ∞.

Typical dose-response curves for these three models are shown in Figure 1.

The **Category **model uses indicator variables defined by cutpoints 0 < γ_1 _< γ_2_

dosecat1 = 1 if 0 < D_φ _≤ γ_1 _and = 0 otherwise

dosecat2 = 1 if γ_1 _< D_φ _≤ γ_2 _and = 0 otherwise

dosecat3 = 1 if γ_2 _< D_φ _and = 0 otherwise

The Control model is nested by β_1 _= β_2 _= β_3 _= 0. D_φ _= 0 also defines the baseline. The model is applied here only with latencies obtained from the two-phase model, and cutpoints γ_1 _= 0.025 (0.25 mSv), γ_2 _= 0.05 (0.5 mSv) which assign roughly equal p-y to dosecat1, dosecat2, and dosecat3.

When φ = 5 years, dosecat1 specifies 699 cells (620850.09 p-y). Hiroshima data is contained in 544 cells (573199.23 p-y), while 155 cells (47650.86 p-y) contain Nagasaki data. Mean attained age ranges from 8.16 years to 96.84 years, and mean age-at-exposure ranges from 1 to 82.99 years. All individual doses within these cells are below 5 mSv. The weighted adjusted colon dose for the cell ranges from 0.004 mSv to 0.25 mSv. As φ increases, cells move to the baseline. For example at φ = 36.9 years, dosecat1 specifies 156 cells (104592.3 p-y) of which 115 cells (95871.94 p-y) refer to Hiroshima and 41 cells (8720.36 p-y) to Nagasaki.

Likewise when φ = 5 years, dosecat2 specifies 560 cells with 519832.84 p-y, of which 109 cells (95907.76 p-y) contain Hiroshima data and 451 cells (423925.08 p-y) contain Nagasaki data. Mean attained age ranges from 8.21 years to 93.32 years and mean age-at-exposure ranges from 0.92 to 84.71 years. All individual doses within these cells are below 5 mSv. The weighted adjusted colon dose for the cell ranges from 0.25 mSv to 0.49 mSv. At φ = 36.9 years, dosecat2 specifies 115 cells (97536.25 p-y) of which 28 cells (21631.85 p-y) refer to Hiroshima and 87 cells (75904.4 p-y) to Nagasaki.

Finally when φ = 5 years, dosecat3 specifies 1579 cells with 548454.05 p-y, of which 780 cells (359098.67 p-y) contain Hiroshima data and 799 cells (189355.38 p-y) contain Nagasaki data. Mean attained age ranges from 8.12 years to 102.2 years and mean age-at-exposure ranges from 0.93 to 91.56 years. All individual doses within these cells are below 20 mSv. The weighted adjusted colon dose for the cell ranges from 0.5 mSv to 20 mSv. At φ = 36.9 years, dosecat3 specifies 353 cells (97629.42 p-y) of which 176 cells (63005.21 p-y) refer to Hiroshima and 177 cells (34624.21 p-y) to Nagasaki.

Note that whilst the dosecat variables are well defined on the cells, they have no simple interpretation in terms of individual doses within those cells. A cell with D_φ _≤ 0.025 (0.25 mSv) may contain individuals with lagged doses up to 5 mSv; it is only the weighted average which is constrained below 0.25 mSv.

Each model is also constrained by λ = λ_0_(1 + ERR) ≥ 0 (equivalently ERR ≥ -1) in all cells, as the Poisson distribution is undefined if λ_i_T_i _< 0.

Poisson regression [12] is based on minimising the model Deviance, or equivalently minimising Σ_i _[E_i _- O_i_ln(E_i_)] where E_i _and O_i _are the expected and observed values.in each cell. While O_i _is given by the data, E_i _depends on the model and its parameter values. The data were placed on a spreadsheet and the Excel tool Solver (Newton-Raphson iteration) was used to optimise the parameters subject to defined constraints.

With the control model, the minimal Deviance is independent of φ. Once φ is fixed, the linear model has a unique minimum Deviance. With other models, local minima were compared to find the absolute minimum DevMod_φ_. Searches were conducted by partitioning the τ axis as [0,1], [1,2]... [2^j-1 ^,2^j^]... [2^8^,2^9^], [2^9^,∞) and beginning iteration from (C, 0, 0, τ_j_) where C represents the control model Maximum Likelihood Estimate parameter values, β and σ are initially set to 0 (β is constrained to 0 for the transient model); while τ_j _is constrained to the j^th ^interval.

If model *I *is nested within model *J *by k parameter constraints, the likelihood ratio test for comparing the two models at φ, LRT_*J-I*,φ _= DevMod_*I*,φ _– DevMod_*J*,φ _is approximately χ^2 ^distributed on k df. At a given φ, LRT_lin-con _and LRT_trans-con _for comparing the linear and transient models with the control model each have 1 df, while LRT_2p-con _for comparing the two-phase model with the control model has 2 df. Note that LRT_2p-trans_, LRT_2p-lin_, and LRT_trans-lin _have 1 df as each nested pair is defined by a single parameter constraint, and that LRT_2p-con _= LRT_2p-lin _+ LRT_lin-con_.

For each model *J *(linear, transient, and two-phase), I found best fits by choosing φ = φ_m _to maximise LRT_*J*-con,φ _(equivalently to minimise DevMod_*J*,φ_) either absolutely or with the constraint that ERR at D_φ _= 0.025 be ≥ 0. The value of φ_m _may vary with the model.

Profile Likelihood Confidence Intervals for ERR were computed at specified values of D_φ_. For example, in the two-phase model ERR depends on D_φ _and T = (β,σ,τ) whilst Deviance depends on (φ,C,T) where C is the vector of control parameters. With φ fixed, let V_φ _be the set of T which can be ruled out with 95% confidence, as all choices of C give Dev(φ,C,T) – DevMod_φ _> K, where K ~ 7.8147 satisfies χ_3_^2^(K) = 0.95. Define Min_φ _(respectively Max_φ_) as the largest v (smallest w) for which the constraint ERR(D_φ_,T) < v (> w) implies T ∈ V_φ_. The interval (Min_φ_, Max_φ_) is taken as a 95% CI for ERR(D_φ_) in the two-phase model.

## Results

For each cancer site and for φ = 5, 6, ... 44, models were fitted to the 0 – 20 mSv male/female (M/F) data.

For the linear model only, I estimated ERR_0.025,φ _by fitting the model to the restricted subcohort of cells with mean weighted adjusted colon dose ≤ 0.5 mSv; ERR_1,φ _was obtained from fitting the 0 – 20 mSv data, as with all other modelling.

More precise optimal latencies φ_m_, 95% CI's for ERR0.025,φm
 MathType@MTEF@5@5@+=feaafiart1ev1aaatCvAUfKttLearuWrP9MDH5MBPbIqV92AaeXatLxBI9gBaebbnrfifHhDYfgasaacH8akY=wiFfYdH8Gipec8Eeeu0xXdbba9frFj0=OqFfea0dXdd9vqai=hGuQ8kuc9pgc9s8qqaq=dirpe0xb9q8qiLsFr0=vr0=vr0dc8meaabaqaciaacaGaaeqabaqabeGadaaakeaacqqGfbqrcqqGsbGucqqGsbGudaWgaaWcbaGaeGimaaJaeiOla4IaeGimaaJaeGOmaiJaeGynauJaeiilaWIaeqOXdy2aaSbaaWqaaiabb2gaTbqabaaaleqaaaaa@391B@ and ERR1,φm
 MathType@MTEF@5@5@+=feaafiart1ev1aaatCvAUfKttLearuWrP9MDH5MBPbIqV92AaeXatLxBI9gBaebbnrfifHhDYfgasaacH8akY=wiFfYdH8Gipec8Eeeu0xXdbba9frFj0=OqFfea0dXdd9vqai=hGuQ8kuc9pgc9s8qqaq=dirpe0xb9q8qiLsFr0=vr0=vr0dc8meaabaqaciaacaGaaeqabaqabeGadaaakeaacqqGfbqrcqqGsbGucqqGsbGudaWgaaWcbaGaeGymaeJaeiilaWIaeqOXdy2aaSbaaWqaaiabb2gaTbqabaaaleqaaaaa@3561@, and Goodness-of-Fit (GoF) statistics were determined for the nested models, including separate male and female results. The category model (M/F) was then fitted using the optimal latencies from the two-phase model.

Results are now outlined for specific cancers (M/F).

### Stomach

**Figure 2:**
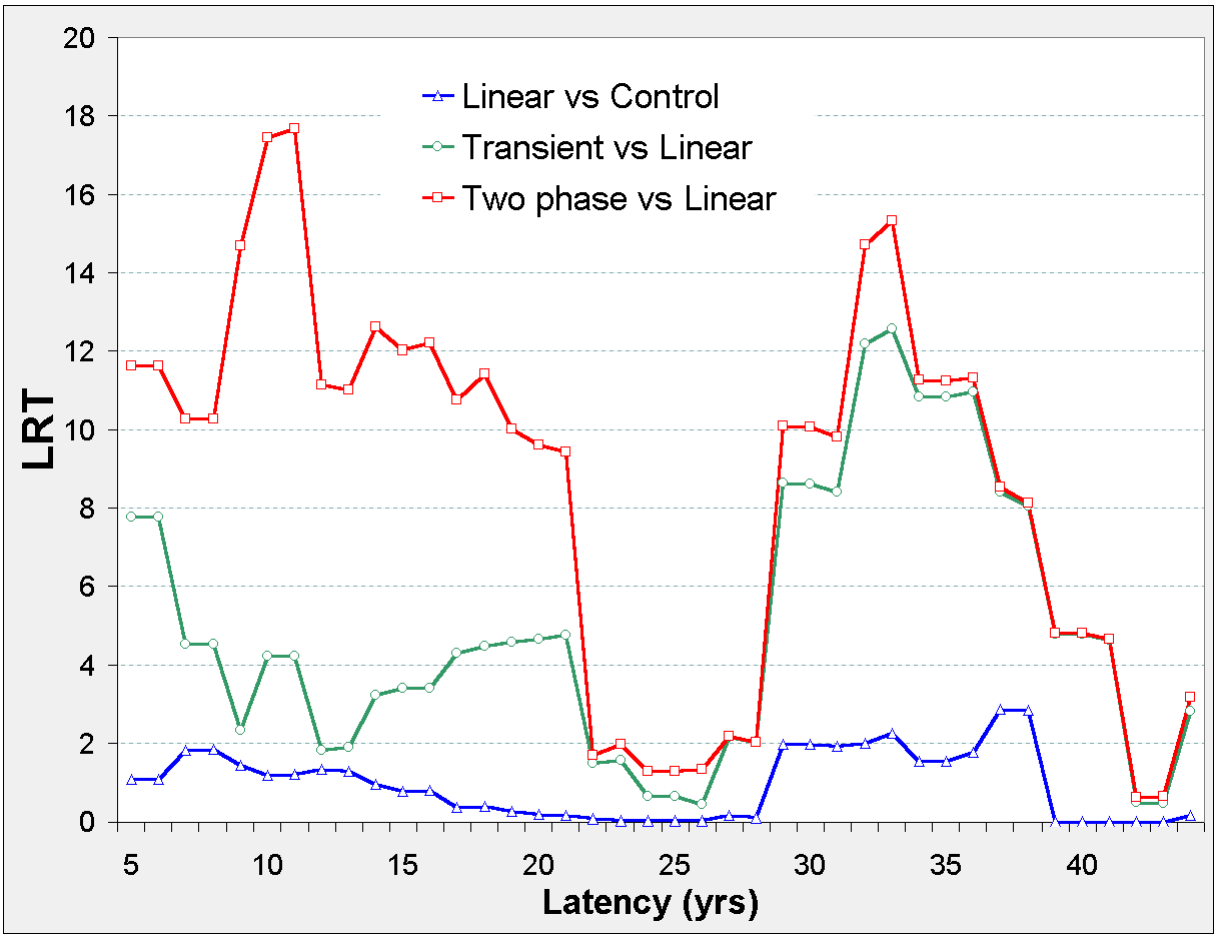
**Stomach cancer mortality in 0 – 20 mSv subcohort, LRT vs latency**. LRT is the likelihood ratio test for comparison of the indicated pair of models evaluated at fixed latency φ, and is χ^2 ^distributed on 1 d.f. (red line shows LRT_2p-lin _for comparing the two-phase and linear models, green line shows LRT_trans-lin _for comparing transient and linear models, blue line shows LRT_lin-con _for comparing linear and control models) LRT values above 3.84 are significant at p = 0.05, above 6.63 at p = 0.01, above 10.83 at p = 0.001. Other LRT can be derived at each φ value, e.g. LRT_2p-con _= LRT_2p-lin _+ LRT_lin-con _and LRT_trans-con _= LRT_trans-lin _+ LRT_lin-con _while LRT_2p-trans _= LRT_2p-lin _- LRT_trans-lin_. Latency φ (in years) is used in defining the lagged dose D_φ _= DS86 weighted adjusted colon dose if Time-Since-Exposure ≥ φ, D_φ _= 0 otherwise. Models specify the Excess Relative Risk ERR as a function of D_φ _while controlling for gender, log attained age, and age-at-exposure categories. Control: ERR = 0 Linear: ERR = βD_φ_; control model nested by β = 0. Transient: ERR = σD_φ _e^(-τDφ) ^with τ ≥ 0; linear model nested by τ = 0. Two-phase: ERR = βD_φ _+ σD_φ _e^(-τDφ) ^with τ ≥ 0; linear model nested by σ = 0; transient model nested by β = 0.

**Figure 3:**
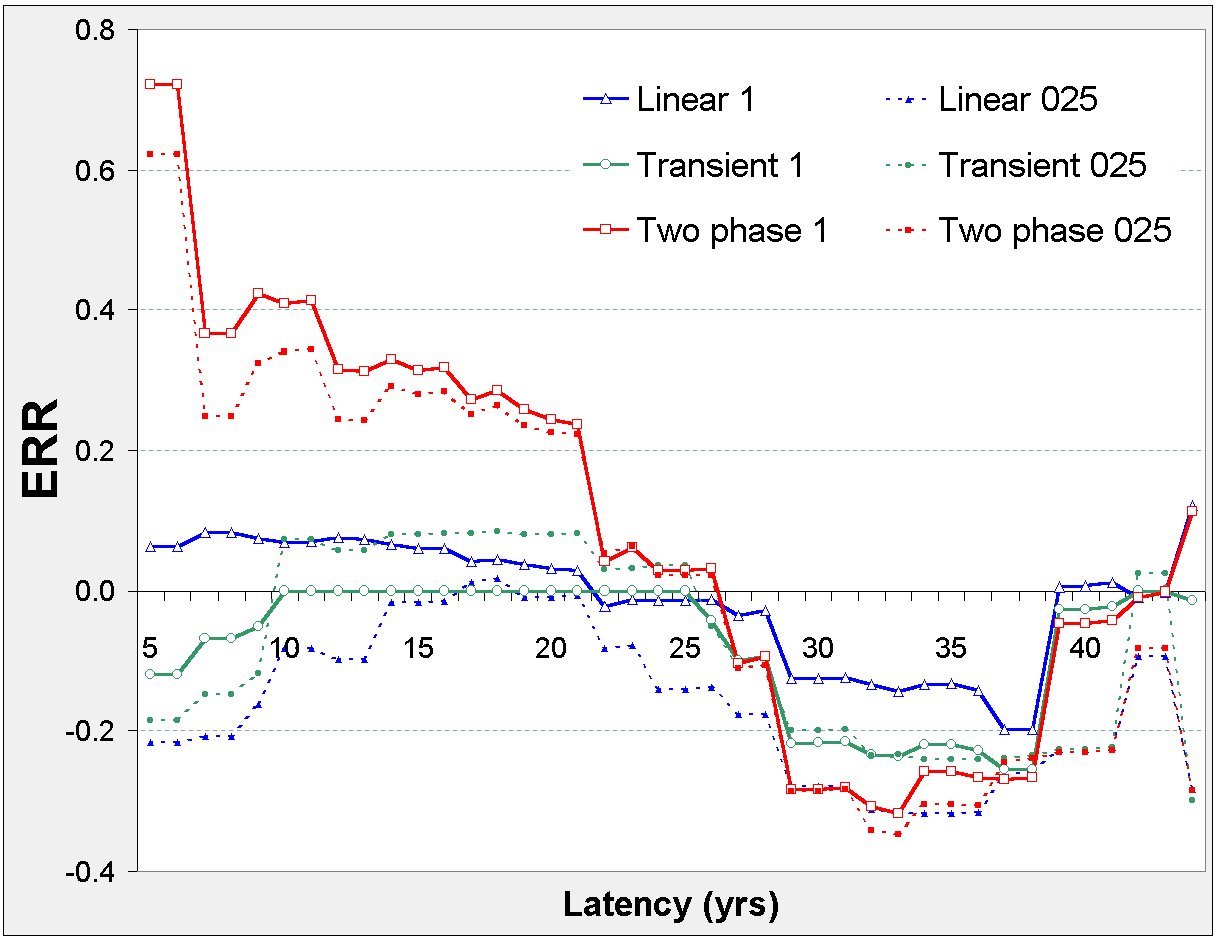
**Stomach cancer mortality in 0 – 20 mSv subcohort, ERR vs latency**. For each model (two-phase in red, transient in green, linear in blue) fitted at fixed latency φ, series 1 (solid lines) shows the Excess Relative Risk ERR_1 _(ERR at 10 mSv lagged dose); series 025 (dotted lines) shows ERR_0.025 _(ERR at 0.25 mSv lagged dose). For latency φ and model definitions see Figure 2. For Linear 025 only, ERR_0.025 _is estimated from the 0 – 0.5 mSv data.

The linear model is never significant against the control model, but Figure 2 suggests two distinct latency periods: 5 ≤ φ ≤ 21, and 29 ≤ φ ≤ 41. Outside these periods, the nested models show no significant improvement on the control model. In both periods, the two-phase and transient models improve on the control model. The two-phase model significantly improves on the transient and linear models when 5 ≤ φ ≤ 21 and on the linear model when 29 ≤ φ ≤ 41. For the two-phase model, ERR_0.025,φ _and ERR_1,φ _are both positive when 5 ≤ φ ≤ 21, and negative when 29 ≤ φ ≤ 41 (Figure 3).

For the linear model (Table 1) φ_m _= 6.66 is optimal in the region of ERR ≥ 0, but the model is not significant against the control model. For the transient model (Table 2) φ_m_= 11.85 is optimal within the range for which ERR_0.025,φ _≥ 0, but ERR is not significantly positive. The optimal latency for the two-phase model (Table 3) is φ_m _= 11.89, when comparison with the control model has LRT_2p-con _= 21.190 (2 df), and comparisons with the transient and linear models have LRT_2p-trans _= 15.684 (1 df) and LRT_2p-lin _= 19.643 (1 df). Each LRT is highly significant. The two-phase model gives ERR_0.025,φ _= 0.391 with 95%CI (0.077, 0.857) while ERR_1,φ _= 0.459 (0.113, 0.942). When D_φ _= Dmax = 0.012, ERR attains a local maximum of 0.539 (0.171, 1.037).

**Table 1:** Linear model^*a*^

	**Stomach**	**Liver**	**Lung**
**gender**	M/F	M/F	M/F
**cases**^*b*^	1482	540	524
**φ**_**m**_^*c*^	*6.66*	38.58	16.90
**ca D**_**φ **_**> 0**^*d*^	1412	172	494
**LRT**_**lin-con**_^*e*^	2.09	11.21	2.26
**ERR**_**1,φ**_^*fg*^	0.09 (-0.03, 0.22)	0.69 (0.25, 1.26)	0.16 (-0.05, 0.42)
**Dev**^*h*^	1680.64	1258.02	1052.43
**Chi-sq**^*i*^	3205.08	2389.85	2500.37
**df**^*j*^	2992	2992	2992
**Excess**^*k*^	38.13	27.17	23.20
			

	**Colon**	**Uterus**	**All solid**

**gender**	M/F	F	M/F
**cases**^*b*^	214	263	4379
**φ**_**m**_^*c*^	*43.91*	*6.12*	43.86
**ca D**_φ _**> 0**^*d*^	33	262	365
**LRT**_**lin-con**_^*e*^	0.19	0.51	5.03
**ERR**_**1, φ**_^*fg*^	0.17 (-0.44, 1.17)	0.10 (-0.16, 0.44)	0.26 (0.03, 0.52)
**Dev**^*h*^	719.80	638.46	2133.03
**Chi-sq**^*i*^	3497.50	1010.56	2769.51
**df**^*j*^	2992	1513	2992
**Excess**^*k*^	1.51	8.88	25.01

^*a *^0 – 20 mSv subcohort, Excess Relative Risk ERR = βD_φ _with β constrained by 1 + ERR ** ≥ **0 for all data cells. D_φ _is the weighted adjusted colon dose (neutron RBE = 10), lagged by φ years, in 10 mSv units. The control model is nested by β = 0.

^*b *^deaths in the subcohort from the specified cancer

^*c *^Latency φ_m _is chosen in the range 5 – 44 years. to minimise linear model Deviance for the given cancer site and gender. Where italicised, φ_m _minimises Dev subject to ERR ≥ 0, equivalent to β ≥ 0

^*d *^deaths in the subcohort from the specified cancer, for which D_φ _> 0 where φ = φ_m_

^*e *^The likelihood ratio test LRT_lin-con _compares the fitted linear model with the control model at φ_m _and is χ^2 ^distributed on 1 degree of freedom

^*f *^ERR_1,φ _is the Excess Relative Risk at D_φ _= 1 with φ = φ_m_

^*g *^95% Profile Likelihood Confidence Intervals are calculated with φ fixed at φ_m_

^*h *^Deviance of the fitted model

^*i *^Pearson Chi-Square of the fitted model

^*j *^Degrees of Freedom of the fitted model

^*k *^difference between Observed (= Expected) cases and the number expected if D_φ _is set to 0 in every cell after fitting the model

**Table 2:** Transient model^*a*^

	**Stomach**	**Liver**	**Lung**
**gen**	M/F	M/F	M/F
**φ**_**m**_^*b*^	*11.85*	36.90	13.60
**ca D**_**φ **_**> 0**	1323	231	506
**σ**^*c*^	77.56	33.51	34.72
**τ**	129.19	3.56	3.78
**LRT**_**trans-lin**_^*d*^	4.49	10.89	14.39
**LRT**_**trans-con**_	5.91	18.12	15.70
**ERR**_0.025,φ_^*e*^	0.08 (-0.11, 0.23)	0.77 (0.18, 1.67)	0.79 (0.20, 2.01)
**ERR**_1,φ_	0.00 (-0.06, 0.16)	0.95 (0.28, 1.99)	0.80 (0.19, 1.99)
**Dmax**^*f*^	0.01	0.28	0.27
**ERR**_Dmax,φ_	0.22 (-0.04, 0.61)	3.46 (1.00, 7.11)	3.38 (0.90, 8.43)
**Dev**	1676.81	1251.10	1039.00
**Chi-sq**	3397.32	2436.33	2728.08
**df**	2991	2991	2991
**Excess**	91.23	95.89	214.39
			

	**Colon**	**Uterus**	**All solid**

**gen**	M/F	F	M/F
**φ**_**m**_^*b*^	*43.98*	*26.91*	43.86
**ca D**_φ _**> 0**	28	127	365
**σ**^*c*^	26.69	275.88	11.89
**τ**	5.98	132.36	3.68
**LRT**_**trans-lin**_^*d*^	3.17	4.48	11.75
**LRT**_**trans-con**_	3.20	4.48	16.78
**ERR**_0.025,φ_^*e*^	0.58 (-0.25, 1.85)	0.25 (-0.09, 1.13)	0.27 (0.07, 0.51)
**ERR**_1,φ_	0.07 (-0.49, 1.71)	0.00 (-0.23, 1.06)	0.30 (0.03, 0.63)
**Dmax**^*f*^	0.17	0.01	0.27
**ERR**_Dmax,φ_	1.64 (-0.60, 6.10)	0.77 (-0.54, 5.35)	1.19 (0.41, 2.06)
**Dev**	716.79	634.49	2121.27
**Chi-sq**	3473.93	1051.37	2765.95
**df**	2991	1512	2991
**Excess**	8.24	19.99	78.00

^*a *^0 – 20 mSv subcohort, Excess Relative Risk ERR = σD_φ _e(−τDφ)
 MathType@MTEF@5@5@+=feaafiart1ev1aaatCvAUfKttLearuWrP9MDH5MBPbIqV92AaeXatLxBI9gBaebbnrfifHhDYfgasaacH8akY=wiFfYdH8Gipec8Eeeu0xXdbba9frFj0=OqFfea0dXdd9vqai=hGuQ8kuc9pgc9s8qqaq=dirpe0xb9q8qiLsFr0=vr0=vr0dc8meaabaqaciaacaGaaeqabaqabeGadaaakeaacqqGLbqzdaahaaWcbeqaaiabcIcaOiabgkHiTiabes8a0jabbseaenaaBaaameaacqaHgpGzaeqaaSGaeiykaKcaaaaa@358E@ with τ ** ≥ **0 and σ,τ constrained by 1 + ERR ≥ 0 for all data cells; for D_φ _see Table 1. The linear model is nested by τ = 0; the control model is nested by σ = 0.

^*b *^chosen in the range 5 – 44 years. to minimise transient model Deviance; where italicised φ_m _minimises Dev subject to ERR ≥ 0, equivalent to σ ≥ 0.

^*c *^At φ_m_, σ and τ are the fitted Maximum Likelihood Estimate parameter values.

^*d *^At φ_m_, the likelihood ratio test LRT_trans-lin _compares the transient with the linear model and LRT_trans-con _compares the transient model with the control model. Each LRT is χ^2 ^distributed on 1 degree of freedom.

^*e *^At φ_m_, ERR_D,φ _is the Excess Relative Risk at D_φ _= D, with 95% Profile Likelihood Confidence Interval.

^*f *^At φ_m_, Dmax = 1/τ is the value of D which maximises ERR_D,φ_

^*g *^n/a indicates no lower limit could be calculated with 1 + ERR ≥ 0

Other definitions and total cases as Table 1.

**Table 3:** Two-phase model^*a*^

	**Stomach**	**Liver**	**Lung**
**gen**	M/F	M/F	M/F
**φ**_**m**_^*b*^	11.89	*36.90*	*13.60*
**ca D**_φ _**> 0**	1311	231	506
**β**^*c*^	0.46	1.43	0.44
**σ**	119.96	290.09	37.92
**τ**	82.67	76.82	4.47
**LRT**_**2p-trans**_^*d*^	15.68	16.76	0.34
**LRT**_**2p-lin**_	19.64	27.64	14.73
**LRT**_**2p-con**_	21.19	34.87	16.04
**ERR**_0.025,φ_	0.39 (0.08, 0.86)	1.10 (0.26, 2.37)	0.86 (0.14, 3.34)
**ERR**_1,φ_	0.46 (0.11, 0.94)	1.43 (0.48, 2.95)	0.88 (0.12, 3.36)
**Dmax**^*e*^	0.01	0.01	0.23
**ERR**_Dmax,φ_	0.54 (0.17, 1.04)	1.41 (0.50, 2.86)	3.23 (0.09, 9.39)
**Dev**	1661.53	1234.35	1038.66
**Chi-sq**	3408.01	2447.95	2770.40
**df**	2990	2990	2990
**R**^*f*^	262.62	204.11	86.39
**Excess**	390.00	126.36	224.43
			

	**Colon**	**Uterus**	**All Solid**

**gen**	M/F	F	M/F
**φ**_**m**_^*b*^	20.28	*26.91*	43.86
**D**_φ _**> 0**	184	127	365
**β**^*c*^	-0.74	0.47	-0.62
**σ**	11.32	198.10	13.10
**τ**	2.66	81.12	2.32
**LRT**_**2p-trans**_^*d*^	1.32	1.83	1.14
**LRT**_**2p-lin**_	3.01	6.31	12.90
**LRT**_**2p-con**_	3.54	6.31	17.93
**ERR**_0.025,φ_	0.25 (-0.44, 1.46)	0.66 (-0.10, 2.44)	0.27 (0.05, 0.54)
**ERR**_1,φ_	0.05 (-0.53, 1.15)	0.47 (-0.32, 2.12)	0.27 (-0.04, 0.66)
**Dmax**^*e*^	0.32	0.01	0.35
**ERR**_Dmax,φ_	1.31 (-0.71, 1.51)	0.90 (-0.17, 2.96)	1.59 (0.03, 2.54)
**Dev**	716.45	632.66	2120.13
**Chi-sq**	3339.24	1047.11	2759.48
**df**	2990	1511	2990
**R**^*f*^	-14.36	425.41	-19.98
**Excess**	29.34	45.89	74.59

^*a *^0 – 20 mSv subcohort, Excess Relative Risk ERR = βD_φ _+ σD_φ _e(−τDφ)
 MathType@MTEF@5@5@+=feaafiart1ev1aaatCvAUfKttLearuWrP9MDH5MBPbIqV92AaeXatLxBI9gBaebbnrfifHhDYfgasaacH8akY=wiFfYdH8Gipec8Eeeu0xXdbba9frFj0=OqFfea0dXdd9vqai=hGuQ8kuc9pgc9s8qqaq=dirpe0xb9q8qiLsFr0=vr0=vr0dc8meaabaqaciaacaGaaeqabaqabeGadaaakeaacqqGLbqzdaahaaWcbeqaaiabcIcaOiabgkHiTiabes8a0jabbseaenaaBaaameaacqaHgpGzaeqaaSGaeiykaKcaaaaa@358E@ with τ ≥ 0 and β,σ,τ constrained by 1 + ERR ** ≥ **0 for all data cells; for D_φ _see Table 1. The transient model is nested by β = 0; the linear model is nested by σ = 0 or by τ = 0; the control model is nested by β = σ = 0.

^*b *^chosen in the range 5 – 44 years. to minimise two-phase model Deviance; where italicised φ_m _minimises Dev subject to ERR_0.025,φ _≥ 0, a weaker condition than β, σ ≥ 0.

^*c *^At φ_m_, β, σ and τ are the fitted Maximum Likelihood Estimate parameter values.

^*d *^At φ_m_, the likelihood ratio tests LRT_2p-trans _and LRT_2p-lin _compare the two-phase with the transient and linear models (χ^2 ^on 1 df); and LRT_2p-con _compares the two-phase model with the control model (χ^2 ^on 2 df)

^*e *^At φ_m_, Dmax is the value of D giving a local maximum of ERR_D,φ_

^*f *^**R **= 1 + σ/β

Other definitions as in Table 2

Applying the category model (Table 4) with φ = 11.89 gives LRT_cat-con _= 21.590 (3 df) and both β_1 _and β_3 _are significantly positive.

**Table 4:** Category model^*a*^

	**Stomach**	**Liver**	**Lung**
**gen**	M/F	M/F	M/F
**φ**_**m**_^*b*^	11.89	36.90	13.60
**D**_φ _**> 0**	1311	231	506
**T**_**1**_^*c*^	505007.81	104710.40	452429.89
**T**_**2**_	433148.43	100577.71	389939.77
**T**_**3**_	453123.28	97629.42	408114.01
**LRT**_**cat-con**_^*d*^	21.59	33.84	19.30
**β**_**1**_^*e*^	0.48 (0.21, 0.81)	1.30 (0.68, 2.15)	0.96 (0.17, 2.46)
**β**_**2**_	0.16 (-0.08, 0.46)	0.85 (0.25, 1.75)	1.84 (0.66, 4.08)
**β**_**3**_	0.42 (0.16, 0.75)	1.39 (0.70, 2.34)	1.33 (0.38, 3.11)
**Dev**	1661.13	1235.38	1035.39
**Chi-sq**	3391.69	2451.56	3111.30
**df**	2990	2990	2990
			

	**Colon**	**Uterus**	**All solid**

**gen**	M/F	F	M/F
**φ**_**m**_^*b*^	20.28	26.91	43.86
**D**_φ _**> 0**	184	127	365
**T**_**1**_^*c*^	342887.98	128431.04	24656.43
**T**_**2**_	288767.71	153667.68	21860.18
**T**_**3**_	307331.28	145793.72	21917.87
**LRT**_**cat-con**_^*d*^	1.57	8.08	16.31
**β**_**1**_^*e*^	0.07 (-0.40, 0.95)	0.92 (0.19, 2.08)	0.13 (-0.06, 0.34)
**β**_**2**_	0.32 (-0.31, 1.52)	0.28 (-0.30, 1.29)	0.43 (0.16, 0.74)
**β**_**3**_	0.02 (-0.44, 0.89)	0.52 (-0.08, 1.48)	0.29 (0.06, 0.56)
**Dev**	718.42	630.89	2121.74
**Chi-sq**	3625.26	1038.26	2772.40
**df**	2990	1511	2990

^*a *^0 – 20 mSv subcohort, Excess Relative Risk ERR = β_1_dosecat_1 _+ β_2_dosecat_2 _+ β_3_dosecat_3 _with β_i _constrained by 1 + β_i _≥ 0; dosecat_1 _= 1 if 0 < D_φ _≤ 0.025, and = 0 otherwise; dosecat_2 _= 1 if 0.025 < D_φ _≤ 0.05, and = 0 otherwise; dosecat_3 _= 1 if 0.05 < D_φ_, and = 0 otherwise. The Control model is nested by β_1 _= β_2 _= β_3 _= 0.

^*b *^Latency φ_m _is chosen from the results of the two-phase model (Table 3)

^*c *^At φ_m_, T_j _is the p-y observation for which dosecat_j _= 1, j = 1,2,3

^*d *^At φ_m_, the likelihood ratio test LRT_cat-con _compares the category model with the control model (χ^2 ^on 3 df)

^*e *^At φ_m_, β_i _are the fitted Maximum Likelihood Estimate parameter values, with 95%CI's

Other definitions as in Table 3.

The models pass Goodness-of-Fit tests with Dev, but Pearson Chi-Square exceeds df due to one case in a cell with T = 0.4 p-y. The two-phase model is preferable to its nested alternatives by LRT comparisons, and clearly distinguishes the latency periods as regions of positive and negative ERR. At the optimal latency, **R **= 1 + σ/β= 262.62.

### Liver

**Figure 4:**
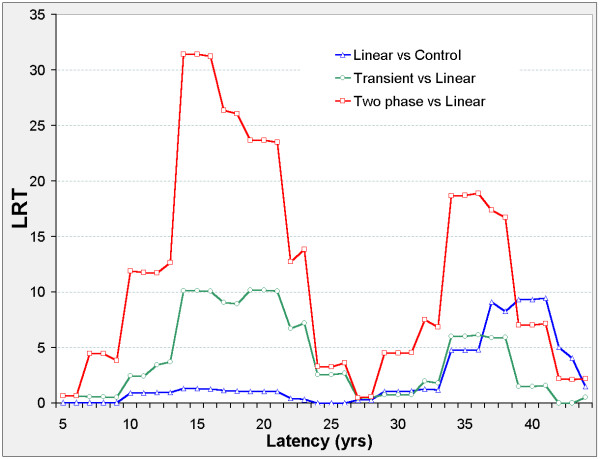
**Liver cancer mortality in 0 – 20 mSv subcohort, LRT vs latency**. Axes and model definitions as in Figure 2.

**Figure 5:**
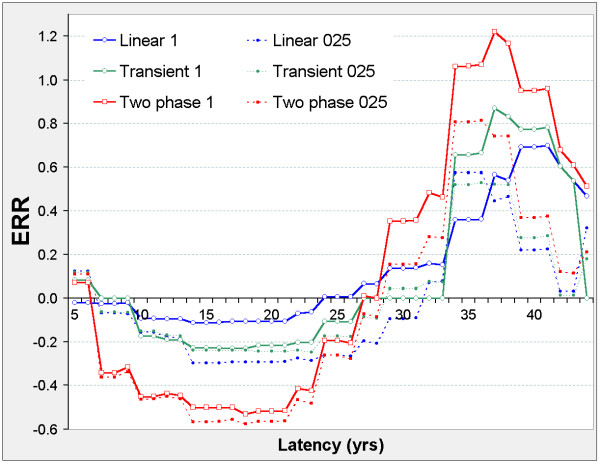
**Liver cancer mortality in 0 – 20 mSv subcohort, ERR vs latency**. Axes and model definitions as in Figure 3.

There are two distinct latency periods outside which the models are insignificant (Figure 4). When 10 ≤ φ ≤ 23 the two-phase model improves on the transient, linear, and control models, and ERR_0.025,φ _and ERR_1,φ _are negative in all three models (Figure 5). When 32 ≤ φ ≤ 41 the two-phase model improves on the transient, linear, and control models, and ERR_0.025,φ _and ERR_1,φ _are non-negative in all three models. When 34 ≤ φ ≤ 43 the transient and linear models each improve on the control model with positive ERR, and when 34 ≤ φ ≤ 38 the transient improves on the linear model.

The optimal latencies are 38.58 (linear), 36.90 (transient), and 36.90 if the two-phase model is optimised with ERR_0.025,φ _≥ 0. At φ_m _= 36.90 the two-phase model has LRT_2p-con _= 34.874 while LRT_2p-trans _= 16.755 and LRT_2p-lin _= 27.642, all highly significant comparisons. At the optimal latency, the two-phase model gives ERR_0.025,φ _= 1.099 (0.264, 2.374) while ERR_1,φ _= 1.428 (0.481, 2.954). The local maximum ERR occurs when D_φ _= 0.013. Estimates from the transient and linear models are comparable.

Applying the category model with φ = 36.90 gives LRT_cat-con _= 33.844 and β_1_, β_2_, and β_3 _are all significantly positive. If "city" is included as a control, LRT_cat-con _= 34.939, β_1 _= 1.35 (0.72, 2.23), β_2 _= 0.73 (0.14, 1.60), β_3 _= 1.39 (0.70, 2.34).

The models pass both GoF tests. The two-phase model is preferable to its nested alternatives by LRT and gives wider periods of significant improvement on the control model, distinguished as regions of positive and negative ERR. At the optimal latency **R **= 204.11.

### Lung

**Figure 6:**
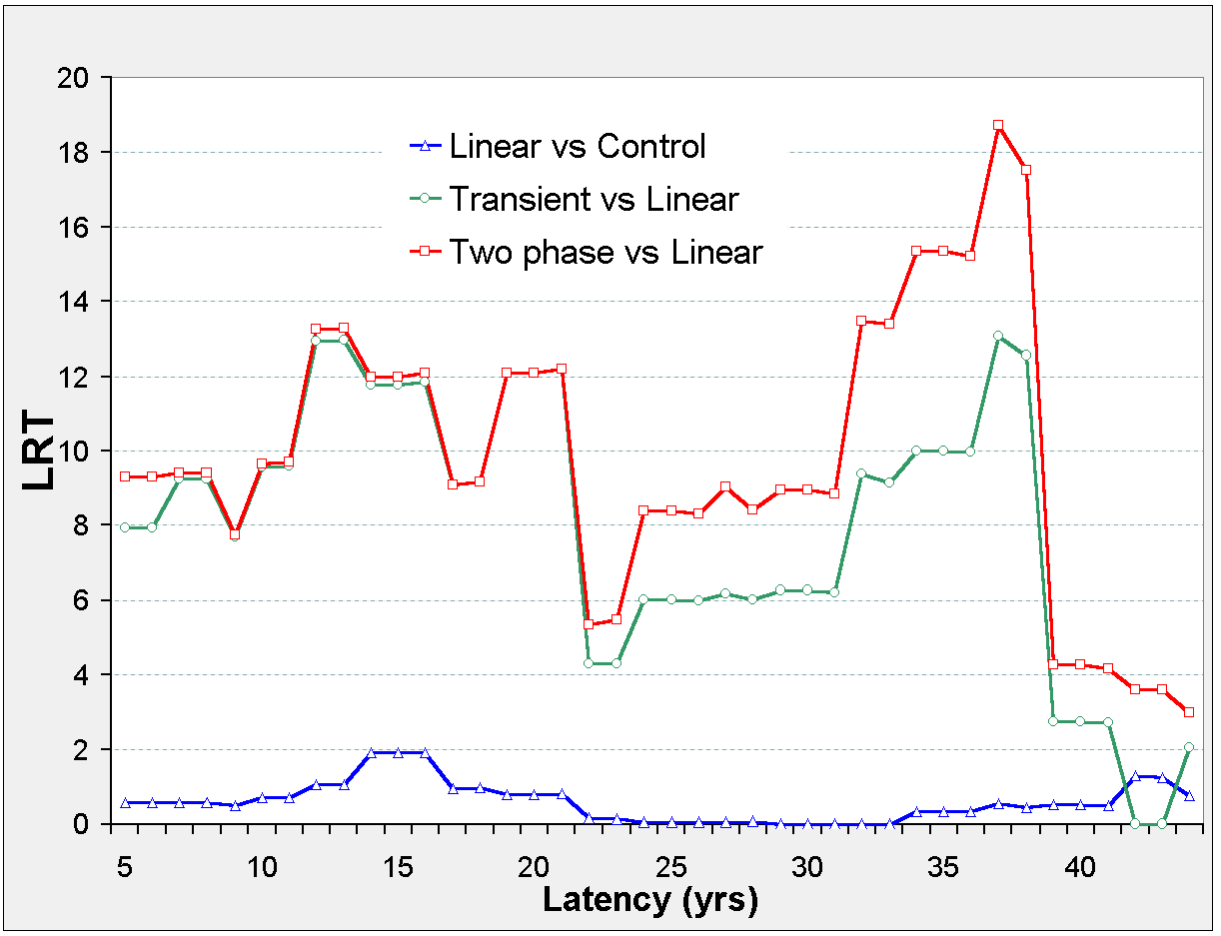
**Lung cancer mortality in 0 – 20 mSv subcohort, LRT vs latency**. Axes and model definitions as in Figure 2.

**Figure 7:**
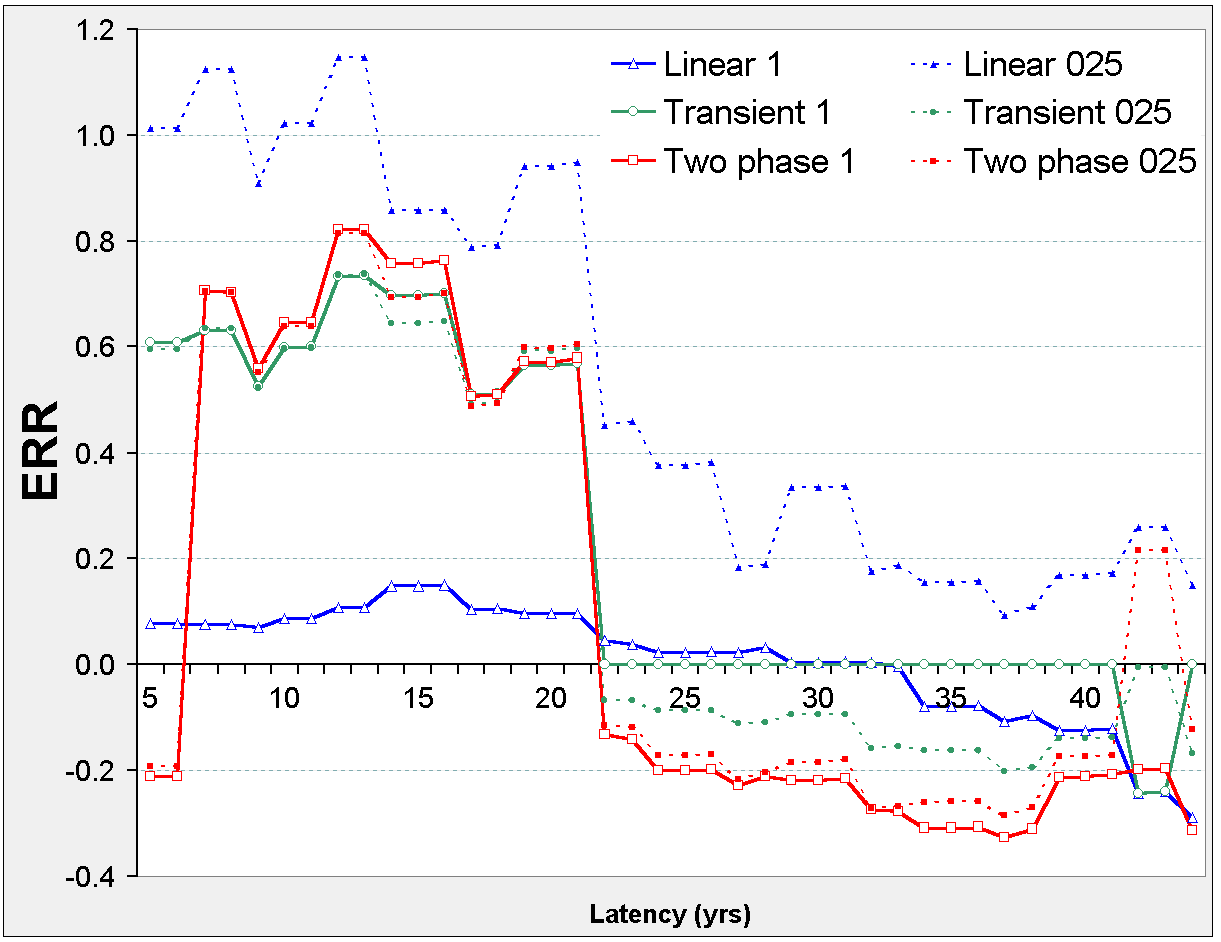
**Lung cancer mortality in 0 – 20 mSv subcohort, ERR vs latency**. Axes and model definitions as in Figure 3.

The linear model is never significant against the control model (Figure 6). The transient improves on the linear and control models when 5 ≤ φ ≤ 38. The two-phase improves on the linear and control models when 5 ≤ φ ≤ 21 and when 24 ≤ φ ≤ 38, and on the transient model when 32 ≤ φ ≤ 38. When 5 ≤ φ ≤ 21 the transient and linear models give positive ERR_0.025,φ _and ERR_1,φ _(Figure 7) as does the two-phase when 7 ≤ φ ≤ 21. When 22 ≤ φ ≤ 41 ERR_0.025,φ _and ERR_1,φ _are negative in the two-phase and transient models.

Optimal latencies are 16.90 (linear), 13.60 (transient), and 13.60 if the two-phase model is optimised with positive ERR_0.025_. At φ_m _= 13.60 the transient model has LRT_trans-con _= 15.701 while LRT_trans-lin _= 14.394, both highly significant. At this latency the transient model gives ERR_0.025,φ _= 0.790 (0.195, 2.006) while ERR_1,φ _= 0.796 (0.189, 1.985). The two-phase model does not improve the transient and gives comparable ERR with somewhat wider CI's. It does improve on the linear model with LRT_2p-lin _= 14.734 and gives **R **= 86.39.

Applying the category model with φ = 13.60 gives LRT_cat-con _= 19.304 and β_1_, β_2_, and β_3 _are all significantly positive.

The 3 nested models pass both GoF tests. The transient model is preferable here.

### Colon

**Figure 8:**
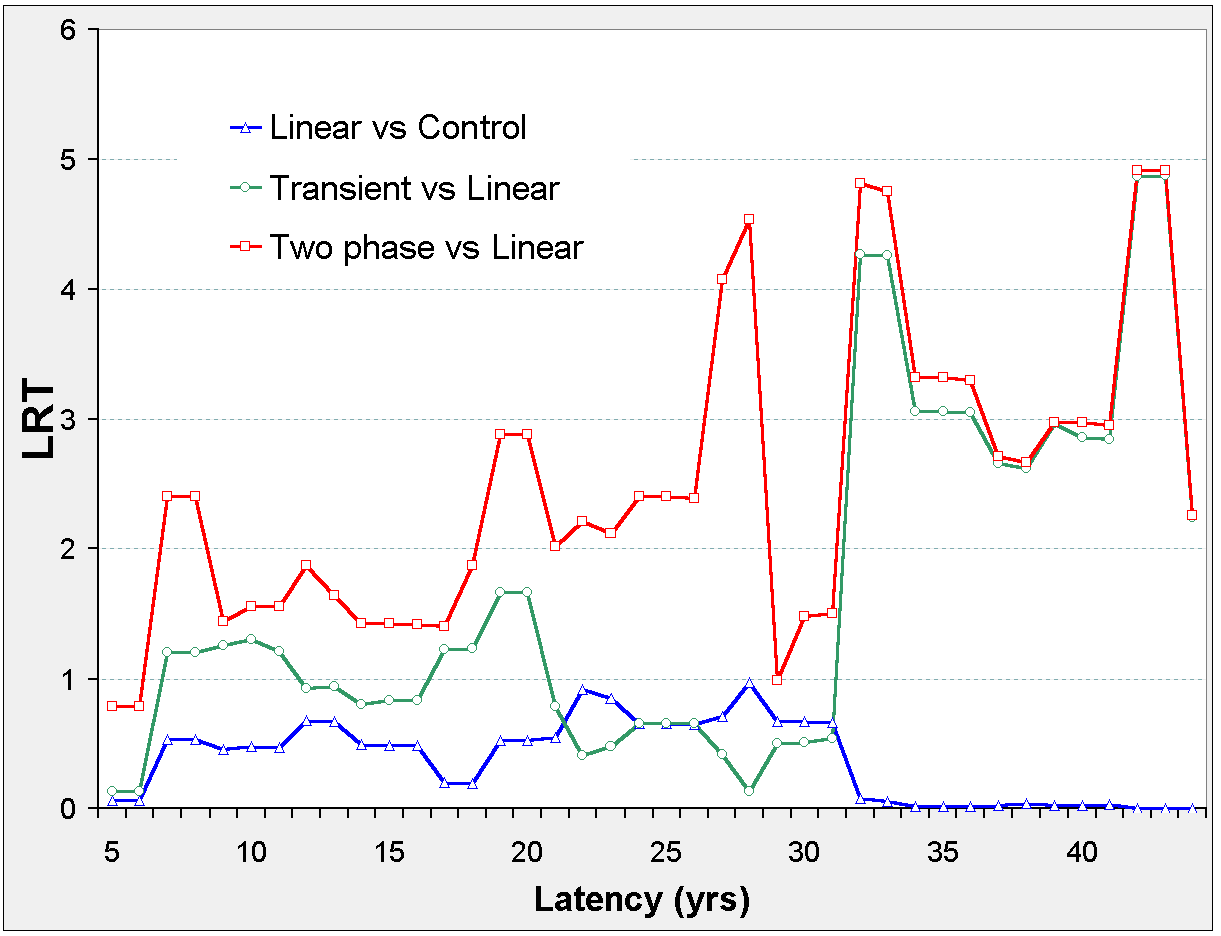
**Colon cancer mortality in 0 – 20 mSv subcohort, LRT vs latency**. Axes and model definitions as in Figure 2.

**Figure 9:**
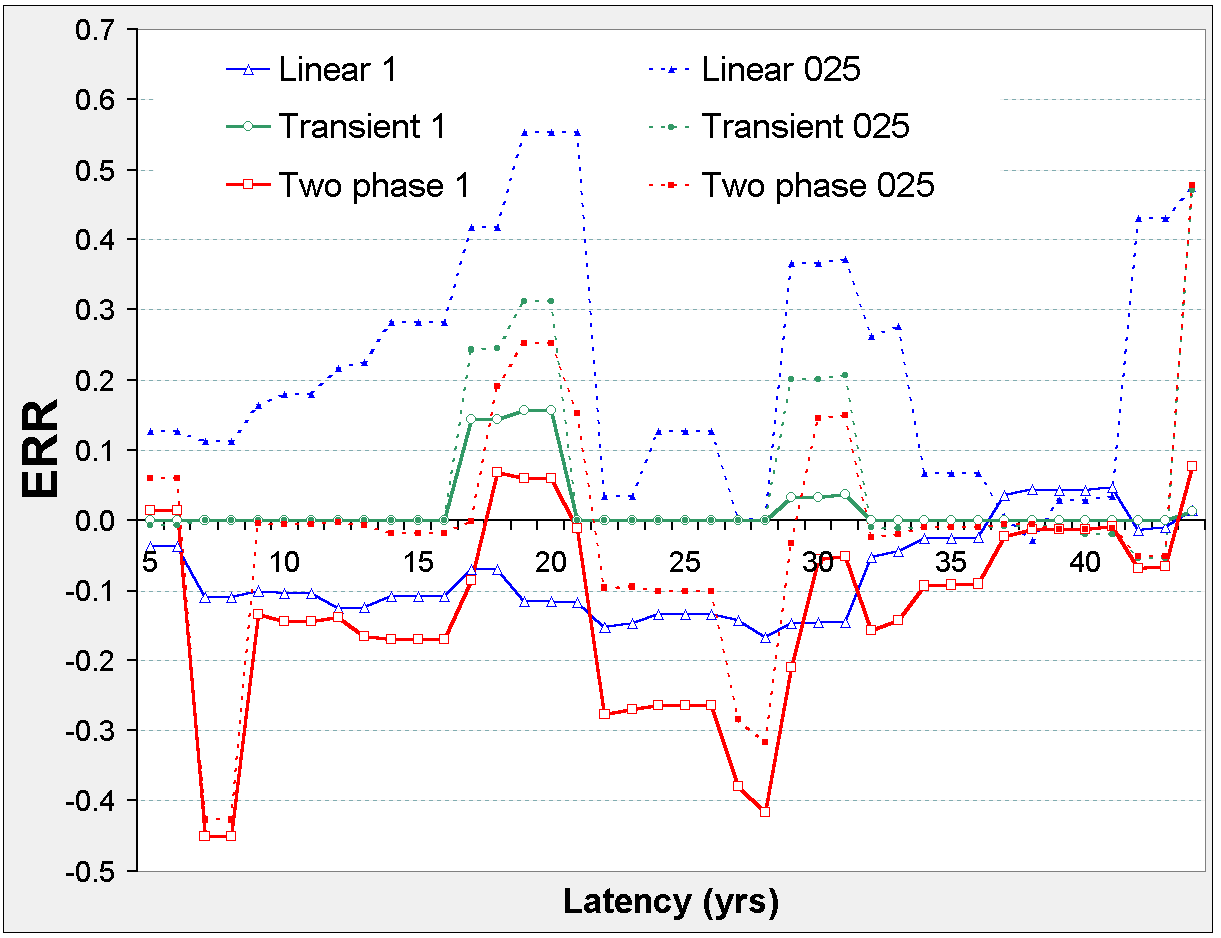
**Colon cancer mortality in 0 – 20 mSv subcohort, ERR vs latency**. Axes and model definitions as in Figure 3.

None of the models give significant positive response for the M/F data (Figure 8, Figure 9). The transient model has occasional weak negative response.

### Uterus

**Figure 10:**
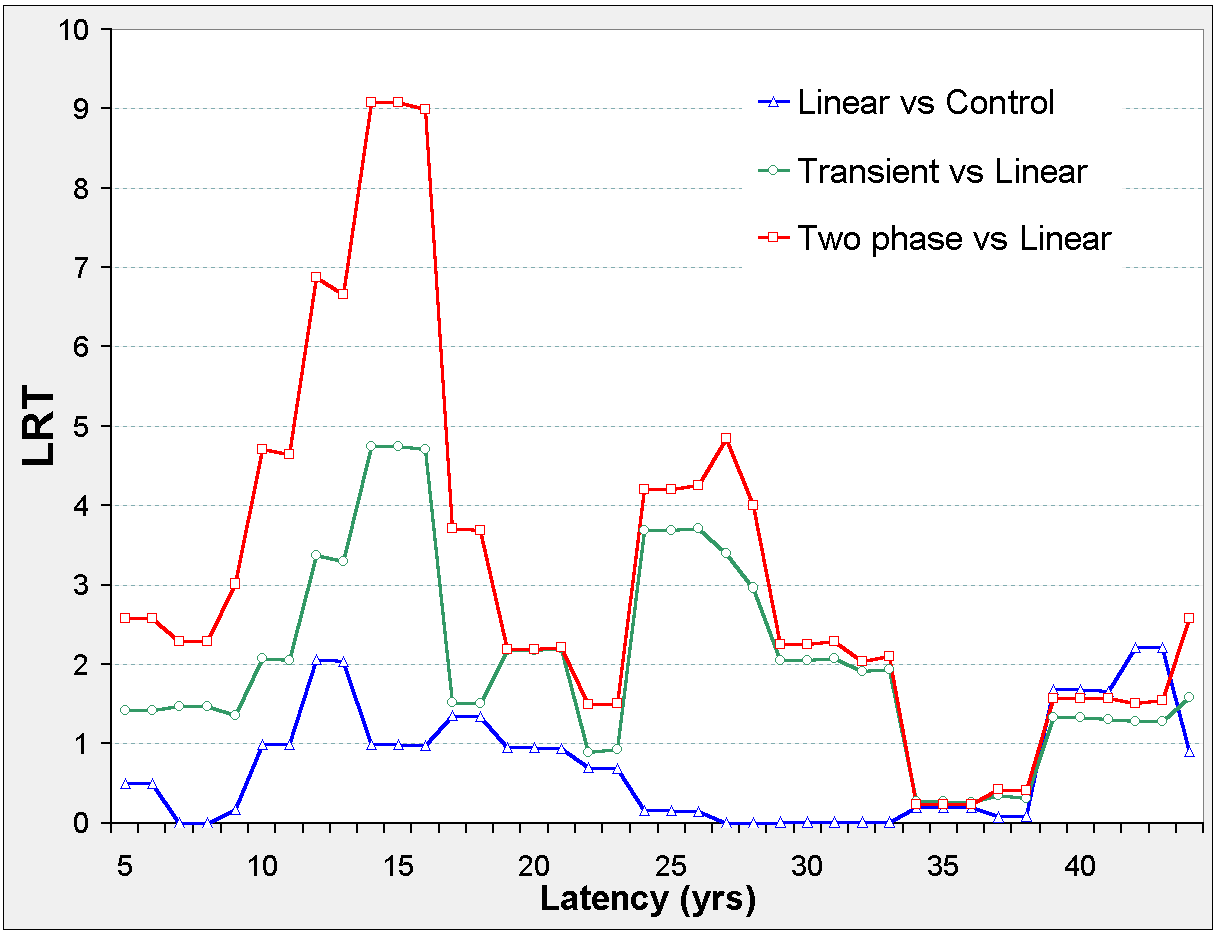
**Uterine cancer mortality in 0 – 20 mSv subcohort, LRT vs latency**. Axes and model definitions as in Figure 2.

**Figure 11:**
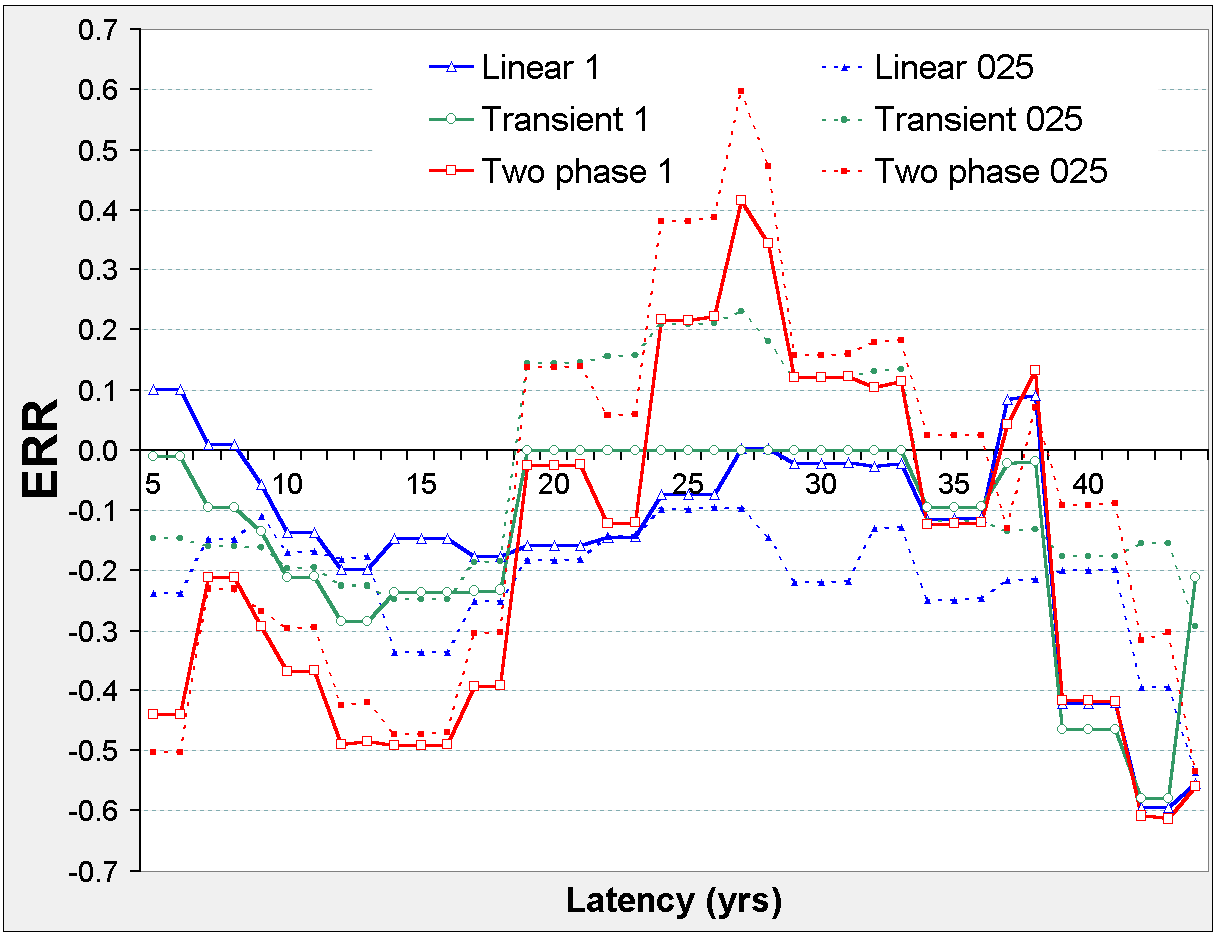
**Uterine cancer mortality in 0 – 20 mSv subcohort, ERR vs latency**. Axes and model definitions as in Figure 3.

The linear model is never significant against the control model (Figure 10). When 12 ≤ φ ≤ 16 the transient and two-phase models both improve on the control model, and ERR is negative in all three models (Figure 11). When 24 ≤ φ ≤ 33 ERR_0.025,φ _and ERR_1,φ _are positive in the two-phase and transient models. At the optimal latency φ = 26.91 (in the region of positive ERR) both the two-phase and transient models are weakly significant against the linear and control models. The 95%CI's do not exclude negative ERR.

Applying the category model with φ = 26.91 gives the weak result LRT_cat-con _= 8.077 with only β_1 _significantly positive.

### All-solid

**Figure 12:**
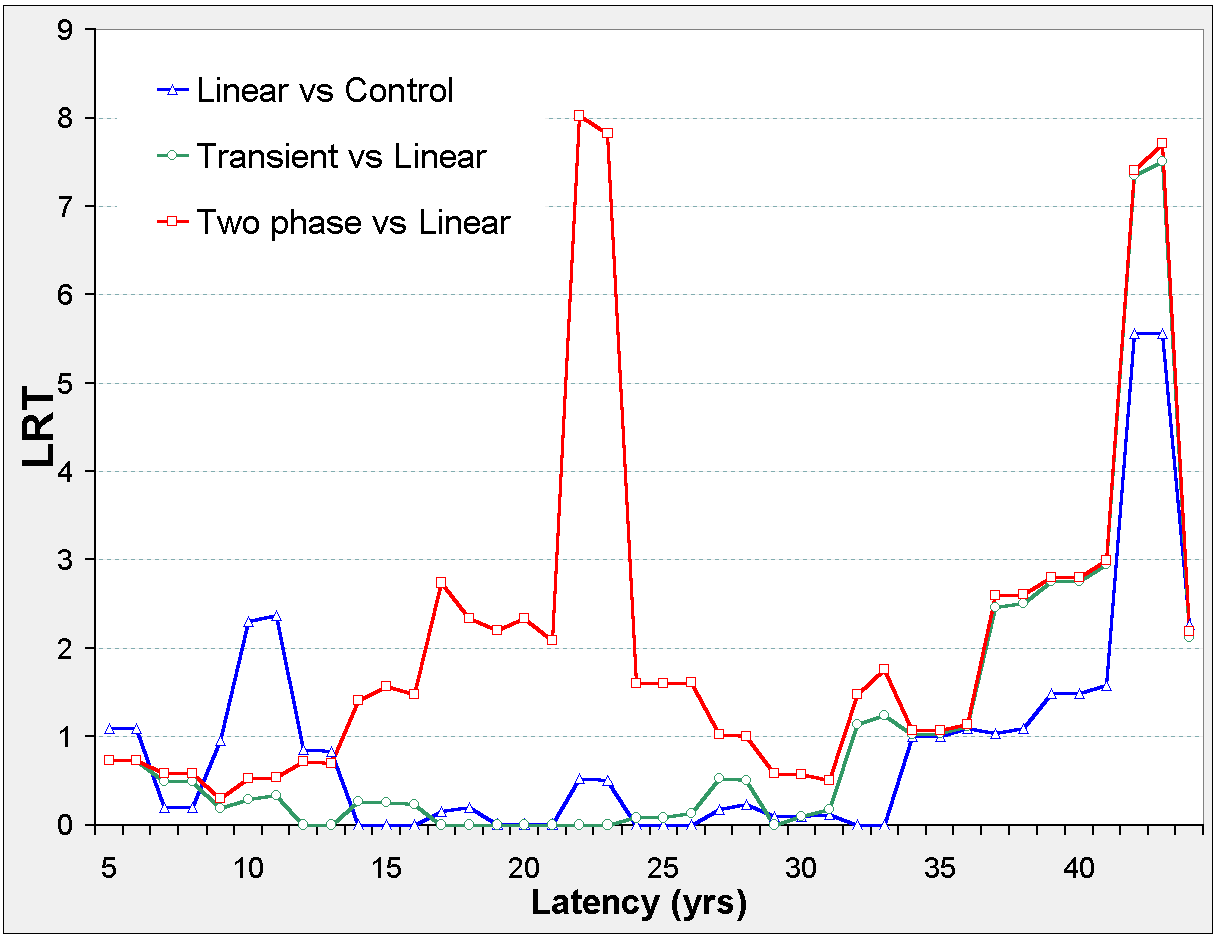
**All-solid cancer mortality in 0 – 20 mSv subcohort, LRT vs latency**. Axes and model definitions as in Figure 2.

**Figure 13:**
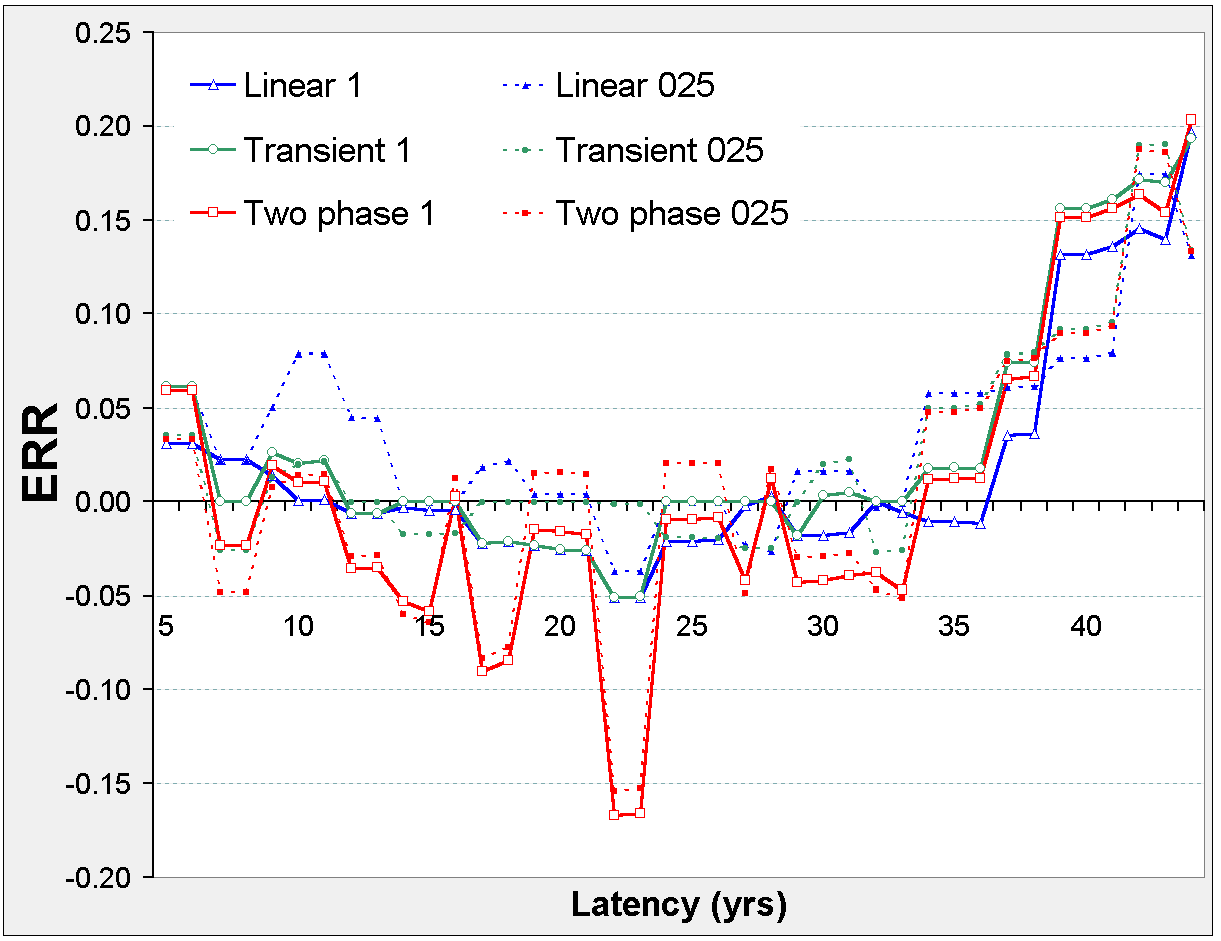
**All-solid cancer mortality in 0 – 20 mSv subcohort, ERR vs latency**. Axes and model definitions as in Figure 3.

When 22 ≤ φ ≤ 23 the two-phase model is significant against transient, linear, and control models (Figure 12) and ERR is negative in all models (Figure 13). When 39 ≤ φ ≤ 44 the transient model improves on the control model, and when 42 ≤ φ ≤ 43 the transient and two-phase improve on the linear and control models. When 37 ≤ φ ≤ 44 ERR is positive in all models. The optimal latency in all three nested models is φ_m _= 43.86, at which all are significant against the control model and the transient and two-phase are significant against the linear model.

At φ_m _= 43.86 the transient model has LRT_trans-con _= 16.781 and LRT_trans-lin _= 11.753, both highly significant, with ERR_0.025,φ _= 0.271 (0.070, 0.509) while ERR_1,φ _= 0.300 (0.034, 0.633). The two-phase model has LRT_2p-con _= 17.925, LRT_2p-lin _= 12.896, both highly significant, while LRT_2p-trans _= 1.143 is not significant. ERR_0.025,φ _= 0.269 (0.051, 0.538) while ERR_1,φ _= 0.272 (-0.044, 0.662). The linear model has LRT_lin-con _= 5.029 and ERR_1,φ _= 0.259 (0.030, 0.522). If applied to the 0 – 0.5 mSv data, the optimal latency is φ_m _= 41.78 at which LRT_lin-con _= 5.562 and ERR_0.025,φ _= 0.175 (0.028, 0.344).

At φ = 43.86 the category model has LRT_cat-con _= 16.311 while β_2 _and β_3 _are significantly positive.

The models pass both GoF tests. The transient model is preferable here, for simplicity.

### Comparison with LSS12

The ERR values found here by applying non-linear models to the 0 – 20 mSv subcohort and optimising latency are several orders of magnitude above those derived by extrapolating from results for a linear model applied with fixed 5 year lag to the entire dose range of A-bomb survivors, as in LSS12 [4] whose Tables AII (Male/Female), AIII (Male) and AIV (Female) show ERR/Sv (organ dose). If a linear model is applied to a single dataset, ERR for d mSv will be (d/1000)(ERR/Sv). Extrapolations of this type are inherent in the ICRP estimate of the risks arising from low doses, which underpin its recommended annual dose limits.

Comparisons are shown in Table 5. Significant discrepancies occur at 10 mSv (ERR_1_) and 0.25 mSv (ERR_0.025_) with the two-phase model for stomach, liver, and lung; and with the transient model for liver and lung.

**Table 5:** Comparison with extrapolations from LSS12^*a*^

**Site**	**gen**	**LSS12 ERR**_1_^*b*^	**2-p ERR**_1,φ_^*c*^	**φ**_**m**_^*d*^	**trans ERR**_1,φ_^*e*^	**φ**_**m**_^*f*^
**Stomach**	M/F	0.0024 (0.001, 0.004)	0.46 (0.11, 0.94)	11.89	0.00 (-0.06, 0.16)	11.85
	M	0.001 (-0.0005, 0.0028)	2.68 (-0.15, 32.45)	6.14	0.00 (-0.21, 0.17)	21.77
	F	0.0047 (0.0022, 0.0077)	0.80 (0.15, 1.77)	13.67	0.43 (0.00, 0.96)	13.68
**Liver**	M/F	0.0037 (0.0013, 0.0065)	1.43 (0.48, 2.95)	36.90	0.95 (0.28, 1.99)	36.90
	M	0.0048 (0.0017, 0.0088)	1.72 (0.40, 4.25)	36.97	1.30 (0.28, 3.10)	36.97
	F	0.0019 (-0.0011, 0.0062)	1.24 (0.06, 3.60)	36.90	1.09 (0.15, 2.78)	38.55
**Lung**	M/F	0.0053 (0.0028, 0.0084)	0.88 (0.12, 3.36)	13.60	0.80 (0.19, 1.99)	13.60
	M	0.0034 (0.0006, 0.0069)	0.78 (-0.11, 4.91)	13.55	0.74 (0.05, 2.40)	13.55
	F	0.0089 (0.0041, 0.0151)	1.35 (0.07, 4.83)	21.91	1.04 (0.09, 3.28)	21.91
**Colon**	M/F	0.0065 (0.0023, 0.0121)	0.05 (-0.53, 1.15)	20.28	0.07 (-0.49, 1.71)	43.98
	M	0.0048 (-0.0002, 0.0127)	0.30 (-0.59, 3.16)	28.63	0.40 (-0.50, 2.72)	28.63
	F	0.0081 (0.0022, 0.0077)	0.47 (-0.55, 2.96)	41.62	0.29 (-0.78, 2.26)	41.62
**Uterus**	F	0.0024 (-0.0006, 0.0067)	0.47 (-0.32, 2.12)	26.91	0.000 (-0.23, 1.06)	26.91
						

**Site**	**gen**	**LSS12 ERR**_0.025_^*b*^	**2-p ERR**_0.025,φ_^*c*^	**φ**_**m**_^*d*^	**trans ERR**_0.025,φ_^*d*^	**φ**_**m**_^*f*^

**Stomach**	M/F	0.00006 (0.000025, 0.0001)	0.39 (0.08, 0.86)	11.89	0.08 (-0.11, 0.23)	11.85
	M	0.001 (-0.0005, 0.0028)	2.43 (-0.23, 31.08)	6.14	0.16 (-0.16, 0.47)	21.77
	F	0.0047 (0.0022, 0.0077)	0.71 (0.05, 1.62)	13.67	0.30 (0.01, 0.76)	13.68
**Liver**	M/F	0.000093 (0.000033, 0.00016)	1.10 (0.26, 2.37)	36.90	0.77 (0.18, 1.67)	36.90
	M	0.00012 (0.000043, 0.00022)	1.30 (0.12, 3.50)	36.97	1.25 (0.25, 3.00)	36.97
	F	0.000048 (-0.000028, 0.00016)	1.03 (0.04, 2.94)	36.90	0.43 (0.01, 1.46)	38.55
**Lung**	M/F	0.00013 (0.00007, 0.00021)	0.86 (0.14, 3.34)	13.60	0.79 (0.20, 2.01)	13.60
	M	0.000085 (0.000015, 0.00014)	0.96 (-0.05, 5.39)	13.55	0.92 (0.12, 2.92)	13.55
	F	0.00022 (0.0001, 0.00038)	1.12 (0.08, 4.15)	21.91	0.91 (0.10, 2.87)	21.91
**Colon**	M/F	0.00017 (0.00006, 0.0003)	0.25 (-0.44, 1.46)	20.28	0.58 (-0.25, 1.85)	43.98
	M	0.00012 (-0.000005, 0.00032)	0.65 (-0.45, 4.34)	28.63	0.72 (-0.30, 3.72)	28.63
	F	0.0002 (0.000055, 0.00019)	0.94 (-0.21, 3.17)	41.62	0.85 (-0.15, 2.56)	41.62
**Uterus**	F	0.00006 (-0.000015, 0.00017)	0.66 (-0.10, 2.44)	26.91	0.25 (-0.09, 1.13)	26.91

^*a *^Life Span Study Report 12 [4]

^*b *^from LSS12 Tables AII, AIII and AIV, scaled by the linear assumption ERR_1 _= ERR per 10 mSv = 0.01(ERR per Sv) and ERR_0.025 _= 0.00025(ERR per Sv)

^*c *^Two-phase model fitted to 0 – 20 mSv subcohort at optimal latency (see also Table 3 for M/F results)

^*d *^Optimal latency for Two-phase model on 0 – 20 mSv subcohort

^*e *^Transient model at fitted to 0 – 20 mSv subcohort at optimal latency (see also Table 2 for M/F results)

^*f *^Optimal latency for Transient model on 0 – 20 mSv subcohort

Estimates of ERR_1 _from the non-linear models are 2 to 3 orders of magnitude above extrapolations from LSS12; for ERR_0.025 _the discrepancy is 3 to 4 orders of magnitude.

### Dosimetry

As a first step towards understanding how dosimetry errors may affect these results, the models were fitted to portions of the 0 – 20 mSv data for the liver. Models were refined to control for "city", although this had little impact. There are 173 data cells with weighted adjusted colon dose = 0. If these cells are deleted from the 0 – 20 mSv subcohort and analysis is restricted to the remaining 2838 cells, fitting the two-phase and linear models for the liver (M/F) at φ = 36.90 years gives LRT_2p-con _= 37.639, LRT_2p-lin _= 30.095, and ERR_1 _= 1.478 (0.52, 3.04). Thus the results for the liver are not caused by any special features of the zero-dose cells.

Alternatively, choose 0 = x_0 _< x_1 _< x_2 _< ... < x_9 _< x_10 _= 20 mSv. Define S_i _as the set of data cells in the 0 – 20 mSv subcohort for which the weighted adjusted colon dose does not fall in the interval [x_i-1_, x_i_]. The x_i _may be chosen so that the S_i _have roughly equal p-y of observation. Then, fitting the two-phase and linear models for the liver (M/F) with φ = 36.9 to the reduced datasets S_i _gives LRT_2p-lin _and ERR_1 _values as shown in Table 6. LRT_2p-lin _values are ≥ 18.94 (1 df) and ERR_1 _is fairly stable, varying from 1.21 to 1.74.

**Table 6:** Two-phase and linear models fittted to subsets of the liver data^*a*^

**i**	**R**_**i**_^*b*^	**p-y in S**_**i**_^*c*^	**Cases**^*d*^	**LRT**^*e*^	**ERR**_**1**_^*f*^
1	0 – 0.01552	1522195.93	441	22.00	1.67
2	0.015521 – 0.019389	1520822.41	459	18.94	1.28
3	0.19396 – 0.022682	1521452.84	499	27.01	1.49
4	0.022687 – 0.025616	1521872.88	500	30.31	1.57
5	0.025619 – 0.30046	1521932.58	510	28.61	1.42
6	0.030066 – 0.037161	1522215.36	494	26.14	1.37
7	0.037211 – 0.87187	1521303.24	483	26.31	1.50
8	0.87209 – 0.97152	1521034.77	492	30.44	1.36
9	0.97154 – 1.0107	1521559.95	507	28.56	1.74
10	1.0108 – 2.000	1519135.79	475	26.42	1.21

^*a *^The two-phase and linear models are fitted to subsets of the 0 – 20 mSv liver data with latency φ = 36.90 years (optimal for the two-phase model on the 0 – 20 mSv data, see Table 3)

^*b *^R_i _is the excluded dose range (in 10 mSv units). S_i _consists of those cells in the 0 – 20 mSv subcohort whose weighted adjusted colon dose does not fall in R_i_

^*c *^Person-years in S_i_

^*d *^Cases (liver cancer deaths) in S_i_

^*e *^LRT = LRT_2p-lin _for comparing the two-phase model with the linear model when fitted to S_i _with latency φ = 36.90 years

^*f *^ERR_1 _is the estimated Excess Relative Risk at 10 mSv when the two-phase model with latency φ = 36.90 years is fitted to S_i_

The DS86 dosimetry used here and in LSS12 was re-investigated by a Joint Working Group using physical measurements, resulting in a new dosimetry system DS02 [11]. A dataset using DS02 and enabling comparison with DS86 was released by RERF last year and can be downloaded as DS02can.dat from the RERF website [9]. However, the cancer mortality fields in this dataset only show deaths from solid cancers (combined), liquid cancers, and leukaemia. Investigation of the stomach, liver, and lung as individual sites is not possible from this public dataset. Furthermore, the stratification of the new dataset differs from that in LSS12 and it is not possible to simply read off the DS02 values for cells in the LSS12 data.

Nonetheless for those cells in the DS02 dataset which have a DS86 dose, the weighted adjusted DS02 colon dose using neutron RBE = 10, shown as "cola02w10", can be compared with the corresponding DS86 dose variable shown as "cola86w10".

Only 11 cells, with 13.83 p-y and no solid cancer deaths, have cola02w10 < 0.005 Sv and cola86w10 ≥ 0.005 Sv. Only 5 cells, with 11.24 p-y and no solid cancer deaths, have cola02w10 ≥ 0.005 Sv and cola86w10 < 0.005 Sv. For cells with cola86w10 ≥ 0.005 Sv the ratio θ = cola02w10/cola86w10 varies from 0.216 to 1.925 with mean 1.08 and standard deviation 0.082. There are only 7 cells, with 10.47 p-y and no solid cancer deaths, for which θ < 0.5. Likewise 92 cells, with 195.84 p-y and 3 solid cancer deaths, have θ > 1.5. In the subcohort with 0.005 Sv ≤ cola86w10 ≤ 0.02 Sv, θ varies from 0.216 to 1.27 with mean 1.026 and standard deviation 0.077. There are 15 cells with 78.5 p-y and 2 solid cancer deaths, for which θ < 0.8, and 9 cells with 9.88 p-y and 1 solid cancer death, for which θ > 1.2

Thus if DS02 is an accurate estimate of the flash dose, there is virtually no misclassification of DS86 between the categories "above 5 mSv" and "below 5 mSv", and above 5 mSv the DS86 dose is a reasonable estimate, though 8% below DS02 on average.

The scope in this paper for misclassification of the dose is therefore reduced if doses below 5 mSv are taken as baseline and doses from 5 to 20 mSv are taken as a single category which is then analysed with latency. While that approach is too crude to detect non-linearity, it gives very similar results for the liver to those found with the linear model.

As shown in Table 1 the optimal latency for the liver (M/F) using the linear model is φ = 38.58, for which comparison with the control model has LRT_lin-con _= 11.21, β = ERR_1 _= 0.69 (0.25, 1.26). Now define E = 1 if Time-Since-Exposure ≥ 38.58 and 5 mSv ≤ colon dose ≤ 20 mSv, E = 0 otherwise. As Time-Since-Exposure is known, errors in E can only arise if doses below 5 mSv were misclasssified as above 5 mSv, or vice versa. Fitting the model defined by ERR = γE to the liver (M/F) data for the 0 – 20 mSv dose range and comparing with the control model (γ = 0) gives LRT = 11.04, γ = 0.67 (0.24, 1.21). Thus the results for the linear model can be reproduced with a two-category model using a cutpoint of 5 mSv, and these categories are almost identical whether defined by DS86 or DS02.

As a further test, the liver data were modelled in the extended DS86 dose range 5 mSv – 500 mSv, where DS86 and DS02 are in reasonable agreement. The two-phase model is a significant improvement on the linear model at latency φ = 36.9 years, with LRT_2p-lin _= 10.37 (1 df) and ERR_1 _= 0.74 (0.10, 1.79).

Similar results for the liver are obtained from the 0 – 500 mSv dose range. The two-phase model is a significant improvement on the linear model at latency φ = 36.9 years, with LRT_2p-lin _= 16.86 (1 df) and ERR_1 _= 0.76 (0.15, 1.66).

For comparison, the results for the liver obtained from the 0 – 20 mSv dose range at latency φ = 36.9 years, are LRT_2p-lin _= 29.67 (1 df) and ERR_1 _= 1.46 (0.50, 3.00). Similar results when "city" is omitted from the model are shown in Table 3.

The DS02 public dataset does not show the liver but does allow modelling of all-solid cancers. The subcohort S defined by cola02w10 ≤ 0.02 and Time-Since-Exposure ≤ 45.39 years (the maximum value in the 0 – 20 mSv subcohort of LSS12 data) has 1682335.39 p-y and 4363 cases, roughly comparable to the LSS12 subcohort analysed with DS86 in this paper. Models were defined as previously, but using cola02w10 in place of the DS86 weighted adjusted colon dose. None of the cells in S contain Nagasaki factory workers. Controls for "city" and "ground distance category" (proximal or distal) were introduced, though they had little impact. If the linear model is fitted to S with latency φ = 43.29 years then LRT_lin-con _= 3.79 (1 df). For the two-phase model LRT_2p-con _= 10.49 (2 df) and LRT_2p-lin _= 6.70 (1 df), again showing non-linearity. ERR_1 _= 0.223 (-0.07, 0.58). For the DS86 results, Table 3 shows ERR_1 _= 0.272 (-0.04, 0.66) at latency 43.86 years.

The estimates of ERR_1 _for all-solid cancers are similar whether derived from the 0 – 20 mSv subcohort of LSS12, or the comparable 0 – 20 mSv subcohort of the DS02 data.

The DS02 data contains an additional 10 years of follow-up. Non-linearity is still present in the extended cohort. Define the subcohort T by cola02w10 ≤ 0.02 without restricting Time-Since-Exposure. At latency 43.29 years the linear model is indistinguishable from the control model (LRT = 0.47) but the two-phase model has LRT_2p-con _= 9.49 (2 df) and LRT_2p-lin _= 9.02 (1 df). ERR_1 _= 0.08 (-0.07, 0.26).

## Discussion

This paper begins from the publicly available data, and I do not know if a comparable analysis of the anonymous individual data would show similar results.

In re-analysing portions of the 1950–90 grouped data, the approach here has four features.

1) To predict risks at 10 mSv, the 0 – 20 mSv data is analysed directly.

2) A variable lag period is used to analyse latency.

3) Dosimetry data is not reduced to categories before modelling. Dose is taken as a numerical variable, defined on the grouped data cells, but results are also tested in a 4-category model with baseline defined by D_φ _= 0 and cutpoints which roughly equipartition the p-y in non-baseline categories.

4) Linearity of the dose response is tested by nesting within more complex models.

With a fixed 5 year lag, none of the cancers considered here show significant effects in the 0 – 20 mSv dose range using a linear model. Allowing latency to vary in this model gives significant positive responses for the liver and all-solid cancers.

Latency reflects biochemical changes required after initial radiation if mutant cells are to progress and form a tumour eventually identified as cause of death, and historical changes in environmental factors which interact with radiation for a particular cancer. Thus latency may be organ and gender specific.

Rothman [13] illustrates how ignoring latency may mask important effects, whether or not the original exposure was brief. Analysis using the lagged dose D_φ _is a simple approach depending on only one parameter. The response might be clearer by modelling the effect of D*w, with w some more general function of Time-Since-Exposure. Such models have been applied to lung cancer mortality in uranium miner cohorts [14,15].

If the linear model were appropriate throughout the low dose region, we might expect ERR_0.025,φ _~ 0.025(ERR_1,φ_) whatever data were used to estimate each ERR. In fact, the ERR values are often comparable. Two non-linear models give significant improvements in the 0 – 20 mSv dose range for the stomach, liver, lung, uterus and all-solid cancers, and for various gender specific sites. These improvements are strong, for example p < 0.001 when comparing the two-phase and linear models (M/F) for stomach, liver, lung, and all-solid; and p < 0.000001 for the liver.

Unlike the linear and control models, the transient and two-phase models require extensive computation as the Deviance may have multiple local minima at any choice of latency φ (fixed when fitting the model). Computation involves a search for local minima, selection of the minimum Deviance at φ, and then a comparison amongst these minima for different φ values. The optimal φ is chosen to give the absolute minimum Deviance, with or without the constraint that ERR_0.025 _be non-negative. The search is streamlined by restricting φ to 5, 6, ... 44 and later refined to consider all φ (to 2 decimal places) in a range which appears likely to contain the optimal value.

I do not know of any general analytical method which might limit the total number of local minima at fixed φ in this data. Instead, the τ axis is partitioned (see Methods). Fitting the model with τ constrained to an interval typically yields a τ value at either endpoint, reflecting the constraint, except for those intervals which contain τ values at which the Deviance attains a local minimum.

Searches begin from the control parameter values which optimise the control model, while τ is confined to the relevant interval and β,σ are initially set to 0. Conceivably, this choice of initial conditions may cause the Newton-Raphson iteration to miss some local minima, though testing other initial conditions did not detect any other solutions. In any case the minimum Deviance at any particular φ can be no higher than the values found here, so any missing minima could only strengthen the evidence of non-linearity.

Fitting the two-phase model to the lung data illustrates these issues. When φ = 13 three local minima are detected. At τ = 4.51, Dev = 1040.35. At τ = 13.86, Dev = 1040.87. At τ = 175.63, Dev = 1050.25. The minimum Dev at φ = 13 is thus 1040.35. The linear model has Dev = 1053.64. Thus LRT_2p-lin _for comparing the two-phase and linear models is 1053.64 – 1040.35 = 13.29, and it is this value which is displayed in Figure 6 when φ = 13. Likewise the ERR value computed at this minimum Dev is displayed in Figure 7 when φ = 13. The resulting graphs indicate the region to be searched for an optimal choice of φ, subject to the constraint ERR ≥ 0. This optimum is φ = 13.6, the value shown in Table 3. At this latency there are again 3 local minima, two of which have similar Dev. At φ = 13.6 the minimum at τ = 4.45 with Dev = 1038.58 has ERR_1 _= 0.88 while the local minimum at τ = 13.54 with Dev = 1039.26 has ERR_1 _= 1.10. Note that while τ varies widely without appreciable change in Dev, ERR_1 _is much more stable. In this example, the 95%CI is (0.12, 3.36). For this reason, ERR is a much better focus for analysis than the model parameters themselves.

Confidence intervals for ERR are often somewhat wider in the two-phase model than in the simpler transient model which is sufficient to describe the lung and all-solid (M/F, F) data. However, as well as improving the fit for the stomach, liver and all-solid (M) the two-phase model gives a more coherent account of the latency regions of significant positive or negative dose response.

Linear extrapolation of the LSS12 results shows almost no response at the doses considered here. Whilst LSS12 uses organ doses and a different system of controls, these factors do not account for the large discrepancy in risk estimates. Alternative controls affect the estimates by a factor of 2 or less, and the use of organ doses has even less impact. Significant discrepancies arise when the dose range is restricted to 0 – 20 mSv and latency is included in the analysis.

Stewart and Kneale [16] found evidence of selection bias in the LSS 1950 – 1985 cohort, thought to reflect the fact that only those victims able to survive from 1945 – 1950 were eligible to enter the cohort. The test group used by Stewart and Kneale to detect bias included less than 4% of the total cohort but had nearly 30% of high doses (> 1000 mSv). It is plausible that such bias would be reduced in the 0 – 20 mSv subcohort, but I cannot test this from the publicly available RERF data.

Pierce and Preston [17] analysed all-solid cancer incidence in Japanese survivors from the 1958 – 1994 tumour registry data for the range 0 – 500 mSv, using a linear ERR model based on colon dose and a categorical model with cutpoints 0, 5, 20, 100, 200, 250, 300, 400 mSv (colon dose). Estimates correspond to ERR ~ 0.006 at 10 mSv. Likewise, if the linear model here is applied to all-solid cancer mortality (1950–90) in the 0 – 500 mSv dose range with latency 5 years, ERR_1 _= 0.004 with LRT = 8.58.

Pierce and Preston focus on survivors who were exposed relatively near the hypocentres of the A-bombs and exclude distal survivors ( ≥ 3 km distant) on the grounds that they had higher baseline cancer rates and that some lifestyle cancer risk factors correlate with urban-rural distinctions, though cigarette smoking had almost no correlation with estimated dose or distance from the hypocentre. Excluding the distal group lowered the baseline by about 5% in their data. Although that is significant in relation to the estimates of ERR in the RERF studies, it is marginal compared to the ERR values found here with the latency models.

The 0 – 20 mSv subcohort contains many proximal as well as distal survivors (10,159 proximal survivors in the incidence dataset had doses below 5 mSv). The results here may of course reflect other risk factors which may correlate and/or interact with radiation dose, but which could only be approached through the individual data. Investigation of possible confounders should also consider latency and non-linear models such as those analysed here, given their clear superiority to the linear model with fixed 5 year lag, for the low-dose grouped data.

This paper is based on the DS86 dosimetry and the results could reflect dosimetry errors, arising from incorrect estimation of the flash dose or by omitting other radiation sources. The doses received in Hiroshima and Nagasaki include the flash dose (used here), induced radioactivity in building materials or soil which persisted for several weeks, "black rain" which fell in the immediate aftermath of the bombings, natural background radiation, global fallout from atmospheric weapons tests, occupational and medical exposures. The public data does not include any individual occupational or medical exposures. Natural background and global fallout should not be correlated with the exposures arising directly from the bombs in 1945, and would be expected to bias results towards the null. Doses from induced radioactivity and "black rain" could be relevant, but currently available RERF public datasets do not include either of these two additional sources.

Errors in the flash dose itself are unlikely to explain the results. Non-linearity and large values of ERR at 10 mSv persist when the zero-dose data is deleted from the 0 – 20 mSv subcohort and likewise when ten intervals spanning 0 – 20 mSv are used to delete dose ranges from the data.

The DS02 dataset shows that there is virtually no misclassification between the DS86 categories "below 5 mSv" and "above 5 mSv". For the liver at latency 36.9 years, it makes little difference whether dose is taken as a categorical variable defined by the 5 mSv cutpoint in the data stratification, or as a numerical value analysed with the linear model.

DS02 and DS86 are in reasonable agreement above 5 mSv. If datacells with DS86 dose below 5 mSv are excluded from the 0 – 20 mSv dose range, the two-phase model is no longer a significant improvement on the linear model for the liver. However, very similar evidence of non-linearity for the liver arises in the 5 mSv – 500 mSv dose range where DS86 is a reasonable approximation to DS02, and in the 0 – 500 mSv dose range considered by Pierce and Preston [17].

Analysis of solid cancers using DS02 in the 0 – 20 mSv dose range gives estimates of ERR comparable to those derived from DS86.

These various arguments suggest that the results are unlikely to be explained by errors in dosimetry of the flash dose, although DS02 like DS86 is subject to some uncertainty due to random errors in specifying individual location and shielding. Separately, induced radioactivity and "black rain" represent additional doses not reported by DS86 or DS02.

Induced radioactivity appears unlikely to fully explain the results. According to the RERF website [6] "The closer to the hypocentre, the higher was the dose [from induced radioactivity]. Past investigations suggested that the maximum cumulative dose at the hypocentre from immediately after the bombing until today is 0.8 Gy in Hiroshima and 0.3–0.4 Gy in Nagasaki. When the distance is 0.5 km or 1.0 km from the hypocentre, the estimates are about 1/10 and 1/100 of the value at the hypocentre, respectively." The issue was examined in detail in the DS86 Final Report Chapter 6 and an Appendix to this Chapter [8]. The cumulative dose from induced radioactivity decreases exponentially with distance from the hypocentre.

From the DS02 dataset, the minimum distance from the hypocentre amongst cells with DS86 doses below 20 mSv is 2.081 km while for cells with DS86 doses below 500 mSv it is 1.210 km. For the 5 – 500 mSv range the total cumulative impact of induced radioactivity would be below 8 mSv in Hiroshima and below 4 mSv in Nagasaki. Consider the unlikely possibility that induced radioactivity adds 8 mSv to those cells which contain Hiroshima liver cancer deaths, and 4 mSv to those cells which contain Nagasaki liver cancer deaths, while leaving all other cells unaffected. Under this extreme assumption, the two-phase model at latency φ = 36.9 years has solutions with ERR_1 _< 0 but the linear model gives LRT_lin-con _= 11.03 (1 df) and ERR_1 _= 0.029 (0.010, 0.054). Conversely, suppose that induced radioactivity adds 8 mSv to those Hiroshima cells which do not contain liver cancer deaths, and 4 mSv to those Nagasaki cells which do not contain liver cancer deaths, while leaving all other cells unaffected. Then the linear model at latency φ = 36.9 years gives LRT_lin-con _= 4.63 (1 df) and ERR_1 _= 0.016 (0.001, 0.036), which is still 4.3 times higher than the LSS12 estimate. These two extreme assumptions may perhaps provide limits on the scope for induced radioactivity to affect the linear model in the 5 – 500 mSv subcohort, where the corresponding results without the addition of induced radioactivity are LRT_lin-con _= 7.25 (1 df) and ERR_1 _= 0.021 (0.005, 0.044). For the 0 – 20 mSv dose range the total cumulative impact of induced radioactivity would be below 0.08 mSv in Hiroshima and below 0.04 mSv in Nagasaki. Whatever consequence this may have for the two-phase model, its effect on the linear model is negligible.

"Black rain" fell primarily at some distance from the hypocentres. According to the RERF website, "Because of wind, the rain mainly fell in northwestern Hiroshima (Koi-Takasu area) and in eastern Nagasaki (Nishiyama area). The maximum estimates of dose due to fallout are 0.01–0.03 Gy in Hiroshima and 0.2–0.4 Gy in Nagasaki. The corresponding doses at the hypocentres are believed to be only about 1/10 of these values." According to Chapter 6 of the DS86 Final Report [8] the rainfall was concentrated around 3000 m from the hypocentres in both cities. From the DS02 dataset, the maximum distance from the hypocentre amongst cells with DS86 doses above 5 mSv is 2.683 km, somewhat closer to the hypocentres than the main rainfall areas. Maximum doses from rainfall were much lower in Hiroshima, though RERF do not give enough detail to estimate an upper bound for the dose from "black rain" in Hiroshima at distances below 2.683 km. However, if the Hiroshima data for the liver is analysed separately for the 5 – 500 mSv subcohort, the two-phase model remains a significant improvement over the linear model at latency φ = 36.9. LRT_2p-lin _= 5.718 (1 df) and ERR_1 _= 0.674 (-0.06, 1.92) while for the linear model LRT_lin-con _= 7.946 and ERR_1 _= 0.027 (0.007, 0.055).

Compounding the uncertainty over doses from "black rain", individuals may have travelled into a rainfall area even if they were outside it when the bombs exploded. RERF's estimates refer to external doses, omitting any inhaled or ingested radiation. In any case the conclusions are provisional until a dataset for specific solid cancers showing DS02 flash doses, induced radioactivity and "black rain" becomes available. Potential confounding by other risk factors cannot be excluded. The neutron RBE of 10 used here, as in LSS12 and elsewhere, may be inappropriate. Despite all these reservations, which also affect the LSS studies, it is striking that both the 0 – 20 mSv and 5 – 500 mSv subcohorts show non-linear dose response for the liver and the two-phase model gives comparable estimates from both subcohorts for Excess Relative Risk at 10 mSv with latency 36.9 years. "Black rain" and induced radioactivity have quite different impacts within these two subcohorts.

I do not know whether these results and the optimal latencies are specific to the A-bomb cohort. In their study of gamma radiation and mortality in the Oak Ridge workforce, Frome et al. [18] find a 'Low-dose β' value of 2.9 with LRT = 3.12 (p = 0.08) for the combined digestive category using a multiplicative model (without latency) with dose restricted to 0 – 640 mSv. Since their unit dose is 1 Sv this corresponds to ERR_1 _= 0.029.

Non-linear models are applied here without assuming any cellular mechanism, and their success in fitting the cohort data does not prove that any particular cell mechanism operated there. However, the two-phase model adapts and simplifies a model derived by Brenner et al. [19] to explain the 'oncogenic transformation frequency' of cells exposed to broad beam irradiation by α particles:

TF = νq<N> + σ [1-e^(-k<N>)^] [e^(-q<N>)^]

TF is the number of transformed cells per surviving cell (Excess Relative Risk of transformation); ν is the transformation frequency for cells struck directly by exactly one α particle, q the surviving fraction of cells struck directly by exactly one α particle, <N> the mean number of α particles striking each cell in the broad beam irradiation (a dose variable), k the number of cells receiving the bystander signal emitted from a cell struck directly by one or more α particles, and σ the (presumed) hypersensitive fraction of bystander cells which are transformed on receipt of any bystander signal. Note that the first term in the TF expression refers to direct effects, whilst the second term refers to bystander effects. As a function of <N>, TF is approximately linear with slope ν q + σ k at very low doses and approximately linear with slope ν q at higher doses.

The two-phase model is asymptotically linear with slope σ + β at very low doses and asymptotically linear with slope β at higher doses. It generates curves of the same qualitative shape as the TF cell model.

In the cell model, the ratio of asymptotic slopes is **R **= 1+(σ k/ν q). From targetted microbeam experiments on C3H 10T^1^/2 mouse fibroblast cells, Brenner et al. estimate ν = 1.3 × 10^-4^, σ = 6.4 × 10^-4^, and q = 0.8. Thus **R **~ 1 + 6.2 k. In a subsequent paper Brenner and Sachs [20] suggest that k ~ 50 (so **R **~ 311) for the human lung, from modelling dose-rate effects in (male) uranium miners exposed to radon.

In the two-phase model, the ratio of asymptotic slopes is **R **= 1 + σ/β. For the male lung, with optimal latency 13.55 years, **R **= 202.22. For the lung, the two-phase model is not significant against the transient and the confidence region for **R **is infinite and includes negative values. But for the stomach (M/F) **R **= 262.62 with 95% CI (102.41, 975.09), and for the liver (M/F) **R **= 204.11 (58.29, 792.85). Thus the low dose A-bomb data is compatible with a bystander model using roughly comparable values of k, the number of cells receiving the bystander signal.

The flash dose comprised gamma and neutron doses. An appropriate cell model for bystander effects following gamma radiation may differ from that developed for α particles, where the impact of a single track on one cell is quite large. However fission neutrons, like α particles, are high LET (linear energy transfer) and might elicit a similar bystander signal. Induction of genomic instability in unirradiated bystander cells has been demonstrated for neutron irradiation in mice [21].

Non-linear dose response curves can also arise from a hypersensitive population subset [22]. Sharp et al. [23] analysed primary liver cancer mortality in relation to Hepatitis B and C in Japanese A-bomb survivors and found a very strong supermultiplicative interaction between HVC and radiation dose, but only for the high and medium dose ranges. For doses below 18 mSv, there was no significant interaction. However, Sharp et al. did not include latency and truncated all liver doses below 3 mSv. I could not analyse primary liver cancer because the data in r12canc.dat shows all liver cancers. Persistent inflammation including chronic liver disease has been detected in Japanese survivors 40 years after the bombing and correlated with radiation dose [24].

## Conclusion

All the models considered here show unexpectedly large and significant results in the 0 – 20 mSv dose range when a lagged colon dose is taken as the main predictor. Non-linear models allowing for asymptotic effects as dose approaches 0 improve the fit and give a much higher slope for the dose response curve near 0 than at higher doses.

Whether or not analyses of this cohort can be transferred internationally, the results here raise questions in Japan. In any case, significant results for doses below 20 mSv are directly relevant to the current ICRP recommendations limiting annual occupational exposure to 20 mSv (whole body dose). Analysis of the 0 – 20 mSv dose range gives responses several orders of magnitude above extrapolations from LSS12 (and other Life Span Studies).

Low dose effects which depend on latency or are detected by non-linear models can still cause significant risks.

## Declaration of competing interests

The author(s) declare that they have no competing interests.

## Authors' contributions

GD conceived of and designed the study and carried out the statistical analysis.
